# Development of potent Affitin-based bispecific NK cell engagers for the therapy of MSLN-expressing cancers

**DOI:** 10.1016/j.omton.2025.201095

**Published:** 2025-11-19

**Authors:** Tina Briolay, Tacien Petithomme, Hermelyne Gravoueille, Judith Fresquet, Sylvia Lambot, Pauline Cossard, Barbara Mouratou, Agnès Fortun, Karine Bernardeau, Agnès Quéméner, Mike Maillasson, Nicolas Boisgerault, Erwan Mortier, François Davodeau, Frédéric Pecorari, Christophe Blanquart

**Affiliations:** 1 Nantes Université, Inserm UMR 1307, CNRS UMR 6075, Université d'Angers, CRCI2NA, 44000 Nantes, France; 2 Nantes Université, University Angers, INSERM, CNRS, Immunology and New Concepts in ImmunoTherapy, INCIT, UMR 1302/EMR6001, 44000 Nantes, France; 3 Nantes Université, CHU Nantes, CNRS, Inserm, BioCore, US16, Plateforme P2R, SFR Bonamy, 44000 Nantes, France; 4 Cibles et Médicaments des Infections et du Cancer, IICiMed, Nantes Université, UR 1155, 44000 Nantes, France; 5 Nantes Université, Inserm, CNRS, CHU Nantes, SFR Santé, FED 4203, Inserm UMS 016, CNRS UMS 3556, Imp@ct Platform, Nantes, France

**Keywords:** MT: Regular Issue, mesothelin, targeting, non-Ig scaffold, Affitin, bispecific NK engagers, cancer

## Abstract

Well-characterized tumor-associated antigens (TAAs) represent targets of interest in many anticancer therapeutic strategies. Although the use of monoclonal antibodies has led this field for years, the development of smaller molecules, with improved properties, holds great potential for specific anticancer applications. Affitins are small (7 kDa), thermostable, and high-affinity nonimmunoglobulin protein scaffolds derived from archaea. Using ribosome display and next-generation sequencing, we isolated the first Affitins specific for human mesothelin (hMSLN), a TAA overexpressed in many solid cancers and currently targeted in clinical trials. The homodimerization of the most promising Affitin (N13) improved affinity by 60-fold (from 35 nM to 0.57 nM). The high specificity of N13 was demonstrated on cell co-cultures under static or dynamic conditions. This Affitin, monomeric or dimeric, was also stable under a wide range of temperatures and upon repeated freeze/thaw cycles. Finally, bispecific natural killer (NK) cell engagers (BiKEs) composed of an anti-cluster of differentiation 16 (CD16) variable heavy domain of heavy chain (VHH) fused to a monomer or a dimer of N13 Affitins were constructed. Using a cytotoxicity assay, we showed the specific lysis of hMSLN-expressing cells in the presence of BiKE and NK cells, supporting the potential therapeutic application of these affinity agents.

## Graphical abstract

**Figure:**
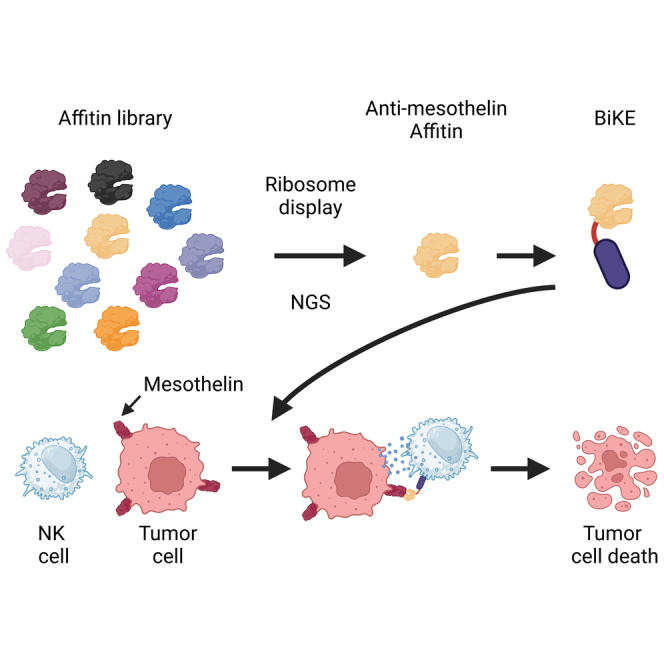

## Teaser

Blanquart and colleagues identified Affitins, derived from extremophilic archaea, for targeting human mesothelin, a well-known tumor-associated antigen. The authors demonstrated their advantageous properties (high affinity, specificity, and stability) and also produced and characterized Affitin-based bispecific NK cell engagers, showing their high efficiency in inducing the death of mesothelin-expressing cells.

## Introduction

Molecules that can specifically bind to cellular targets play an important role as therapeutic and diagnostic agents, especially in cancer medicine. This field of research has been dominated for several years by monoclonal antibodies, which are the most common affinity agents used in cancer research and in the clinic.^1^^,^^2^ The use of antibodies has revolutionized the field of cancer targeting, although these molecules suffer from several drawbacks hindering more widespread application. Indeed, they show limited tissue penetration as a result of their high molecular weight (150 kDa), their production cost is high, and they can be relatively unstable and potentially immunogenic.^3^ As a consequence, several nonimmunoglobulin (non-Ig) protein scaffolds have been proposed with the aim of overcoming these limitations.^3^ These proteins are derived from parent proteins with various physiological functions and are further mutated and selected to enable recognition of targets of interest. Non-Ig scaffolds share several common properties, such as low molecular weight, high stability, relatively low immunogenicity, and low production costs.^3^^,^^4^

Affitins are small (approximately 7 kDa) affinity proteins derived from the Suld7d protein family, such as Sac7d, Sso7d, or Aho7c. These are DNA-binding proteins isolated from the hyperthermophilic archaeon *Sulfolobus* genera.^5^ The randomization of 10–14 residues localized in the DNA-binding site of Sac7d allowed the full redirection of the initial DNA specificity of the protein toward various targets of interest, such as SpA and PulD bacterial proteins, human IgG, and glycosidases.^6^^,^^7^^,^^8^^,^^9^ Recently, Aho7c, a shorter and more stable protein of the Sul7d family than Sac7d, was used to generate specific binders for human epithelial cell adhesion molecule (EpCAM), with dissociation constants in the picomolar range.^10^ Affitins have the particularity of being cysteine-free, single-chain proteins that lack post-translational modifications and are highly stable under a wide range of temperatures and pH.^6^^,^^7^^,^^8^^,^^11^^,^^12^^,^^13^ Thus, Affitins represent an attractive non-Ig scaffold alternative to antibodies and their fragments. In this paper, we propose a tailor-made Affitin as a non-Ig scaffold for the targeting of human mesothelin (hMSLN).

hMSLN is a tumor-associated antigen (TAA) overexpressed in a broad range of solid tumors.^14^^,^^15^ Thus, MSLN has been considered a promising target for the development of targeted therapies against various cancers, notably pleural mesothelioma (PM), an aggressive cancer of the pleura that usually develops after asbestos exposure.^15^^,^^16^ For years, the first-line treatment of PM has consisted of systemic chemotherapy regimens combining a platinum derivate (either cisplatin or carboplatin) and an antimetabolite (pemetrexed).^17^^,^^18^ The recent approval of nivolumab supplemented with ipilimumab for first-line treatment of advanced mesothelioma represents major progress in PM management, although the median overall survival of treated patients remains around 18 months.^19^ Given the urgent requirement to find new therapeutic approaches for patients with PM, several therapeutic alternatives, such as targeted therapy, have been explored.^19^ MSLN is one of the molecular targets most extensively studied in the clinic for PM-targeted therapy. Many phase 2 clinical trials testing MSLN-targeted therapies in PM have been reported and demonstrated the safety of the approach.^16^ Even though the actual clinical efficacy of such approaches is still to be demonstrated in PM, targeting this TAA remains of interest for cancer therapy, as illustrated by encouraging recent results with anti-MSLN chimeric antigen receptor-T-cells (CAR-T) and the ever-growing number of ongoing clinical trials targeting MSLN in various cancers.^16^^,^^20^

Using ribosome display, next-generation sequencing (NGS) followed by clustering analysis, and functional assays, we identified Affitins able to bind to hMSLN in the same region as amatuximab (MORAb-009), an anti-MSLN monoclonal antibody currently evaluated in clinical trials. The homodimerization of the best candidate, resulting in a dimer of 15 kDa, enabled its binding to cellular MSLN on the surface of PM cancer cells, with an affinity of approximately 2 nM. We demonstrated the specificity of MSLN binding by flow cytometry and confocal microscopy in coculture models under either static or dynamic culture conditions. We also characterized the stability of our constructs across a wide range of temperatures and repeated freeze/thaw cycles. Finally, we designed bispecific natural killer (NK) cell engagers (BiKEs) containing a monomer or a dimer of our lead Affitin and validated their capacity to induce specific NK-mediated cytotoxicity against MSLN-expressing PM cells. In this work, we describe the first Affitins capable of binding to hMSLN and demonstrate their potential usefulness for future applications in targeted therapy of MSLN-expressing cancers.

## Results

### Selection of anti-hMSLN Affitins

**Figure 1:**
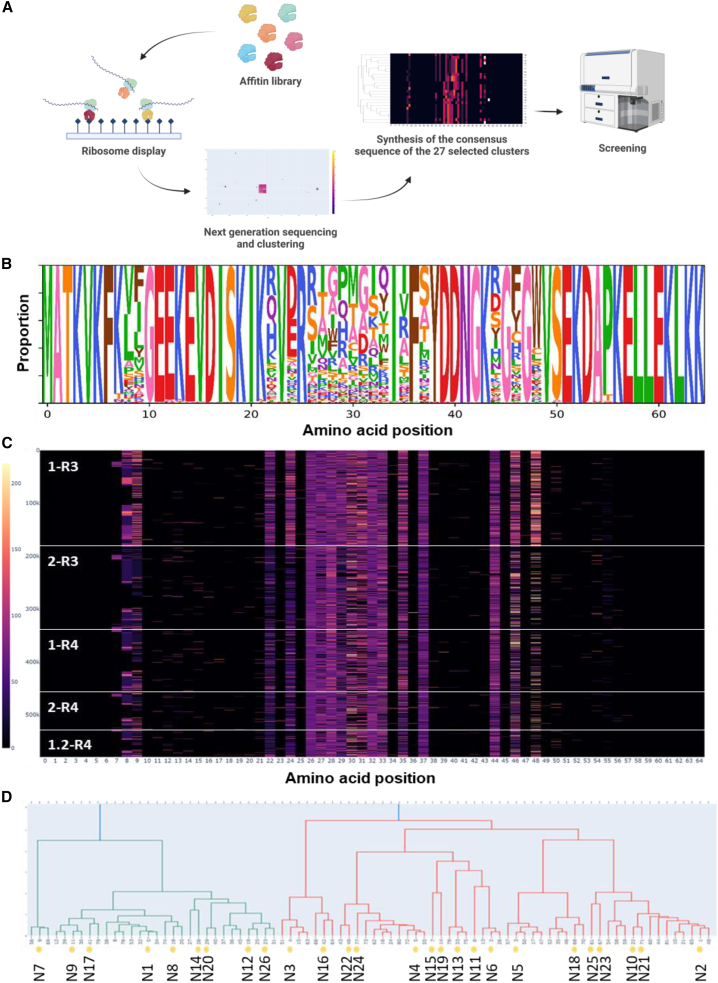
Selection of Affitins specific for human mesothelin (A) Schematic representation of the sequential technical steps that led to the selection of antihuman mesothelin (hMSLN) Affitins. (B) Logo representation of Affitin sequences obtained by NGS. The size of each amino acid letter is proportional to its prevalence at a given position. (C) Heatmap representation of the entire dataset. Each line represents a unique sequence (redundant sequences were removed), and each column corresponds to an amino acid position. Colors represent the distance between an amino acid and the consensus sequence according to the Grantham matrix (see material and methods). White lines delimit sequences from different selection rounds (see Figure S1). (D) Sequences were clustered into 82 clusters, and a consensus sequence was computed for each cluster. Hierarchical clustering was then applied to represent the clusters on a dendrogram. 25 sequences (yellow dots), representing the global variability, were arbitrarily chosen for further characterization.

The aim of our study was to identify and characterize Affitins able to bind to the hMSLN tumor antigen. The general methodology used is described in Figure 1A. We generated two DNA libraries (L5 and L6) of Affitin sequences by PCR. These were transcribed and translated *in vitro*, and several rounds of ribosome display were performed on the entire recombinant hMSLN and on the recombinant 302–359 fragment of hMSLN, alternatively (Figure S1). This fragment of hMSLN contains the epitope targeted by the therapeutic antibody MORAb-009 (amatuximab), which is currently being evaluated in clinic trials.^21^^,^^22^ Elution of Affitin complexes bound to hMSLN was performed as outlined in the diagram in Figure S1. Briefly, Affitins were either eluted with EDTA to harvest all candidates or by competition with an already characterized VHH A1^23^ which binds hMSLN in the same area as the MORAb-009 therapeutic monoclonal antibody. The objective was to harvest only Affitins able to bind to an accessible region of hMSLN on tumor cells. The Affitin pools obtained at the end of the selections were first screened by enzyme-linked immunosorbent assay (ELISA) to evaluate the proportion of binders specific for hMSLN 302–359. After the 3^rd^ round of selection and elution performed with either EDTA (1-R3 in Figure S1) or VHH A1 (2-R3 in Figure S1), we found that 55% and 30% of tested clones were positive in ELISA (i.e., with a specific/aspecific signal ratio per well higher than 10), respectively. For the 4^th^ round, 65% and 88% of the clones were found to be positive in pools corresponding to elution performed with either EDTA (1.2-R4) or VHH A1 (2-R4), respectively. These results show that MSLN-specific Affitins were enriched during selection and suggest that our strategy drove the selection toward epitopes common to the one recognized by VHH A1. Random picking and sequencing of selected Affitins resulted in redundant sequences, suggesting that ribosome display over-amplified a few major sequences. This over-amplification hindered the identification of rare but potentially relevant candidates. To address this issue, the pools of Affitins obtained at the end of selections were subjected to NGS and analyzed using a custom clustering algorithm (see material and methods). Conventional analysis pipelines often emphasize the degree of over-representation of each sequence based on read counts, which overlooks the presence of rare sequences. The level of amplification between each round is influenced not only by protein affinity but also by local sequence context and PCR efficacy, which differ for each sequence. Additionally, ribosome display was performed on a randomized synthetic library, and between each round, retro-transcription may introduce variations in selected binders. Consequently, a consensus binding sequence might not be over-amplified after several rounds of ribosome display, but sequence variations that do not severely alter affinity should be co-selected and still present in the NGS dataset. Thus, our algorithm focuses on unique sequences, disregarding read counts. Over-representation of some sequences was observed in the sequencing data; for instance, although over 60,000 unique Affitin-coding sequences were recovered for the 2-R4 round, about 25% of the reads consisted of a single unique sequence. The diversity and proportion of the sequences obtained after selection rounds are illustrated in Figure 1B (sequence logo representation) and Figure 1C (heatmap, where the color indicates distance to the consensus sequence according to the Grantham matrix). We noticed that elution by competition reduced the number of selected Affitins compared to elution using EDTA, with higher diversity in 1-R3 with EDTA elution compared to 2-R3 with elution by competition. After the 4th round of selection, the main diversity was observed at amino acids 26 to 33, 35, 37, and 44. Sequence clustering revealed 82 clusters, suggesting active selection of these sequence families by ribosome display. Finally, we manually selected 27 sequences among consensus sequences calculated for each cluster based on cluster size and diversity (Figure 1D). Affitins corresponding to these sequences were then produced for characterization (Figure 1D).

### Evaluation of the binding of the 27 candidates to cellular hMSLN

**Figure 2:**
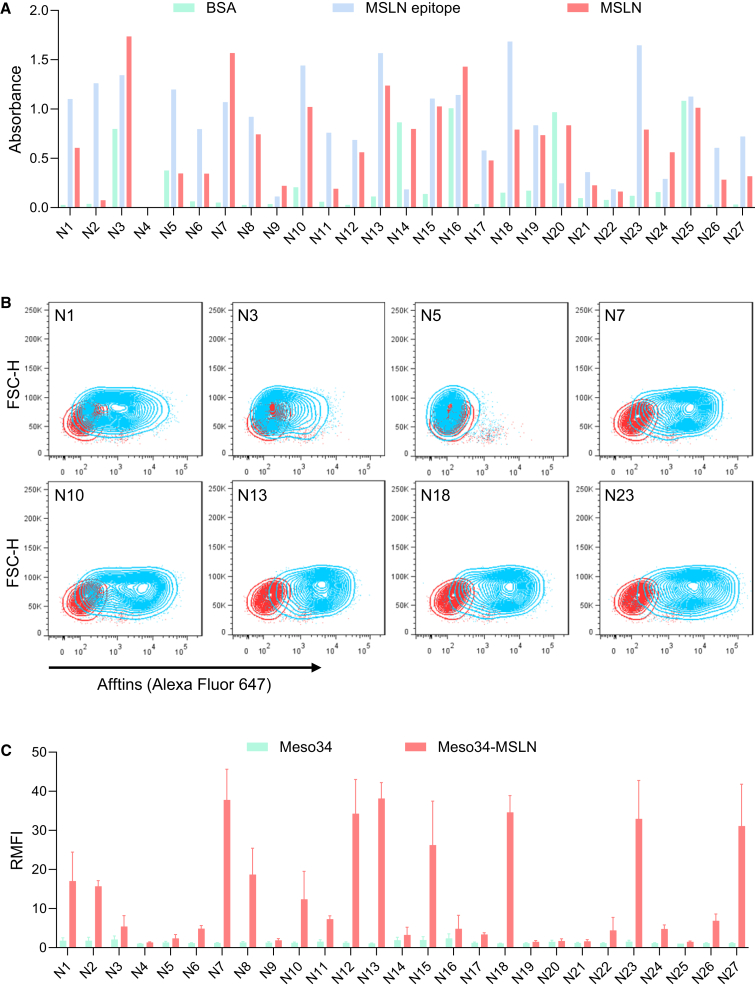
Identification of several Affitins able to bind to recombinant and cellular human mesothelin (A) ELISA of the 27 Affitin candidates (1 μM) against recombinant human mesothelin or the 302–359 fragment of human mesothelin (*n* = 1). (B) The 27 biotinylated Affitins were tetramerized on fluorescent streptavidin to facilitate detection. Meso34 (MSLN−) and Meso34-MSLN (MSLN+) MPM cells were stained with the tetramers, and the binding of the Affitin tetramers was analyzed by flow cytometry. Representative flow cytometry profiles of the experiment are shown in (B). Red: streptavidin AF647 alone; blue: Affitin tetramers. (C) Graph depicting the means of ratios of median fluorescence intensities (RMFI) +/− SEM obtained in the experiment shown in (B), normalized to the control streptavidin signal (*n* = 3 independent experiments).

The first question was to assess whether the Affitins chosen from the NGS analysis were able to recognize hMSLN. The 27 Affitins were produced and purified, with average final yields of 60 mg/L *Escherichia coli* (*E. coli*) culture (16–180 mg/L), and their capacity to bind to the entire recombinant hMSLN or to the recombinant 302–359 fragment of hMSLN was assessed by ELISA (Figure S2A). Most of the candidates were able to specifically bind to the hMSLN 302–359 fragment and to the entire hMSLN (Figure 2A). We next tested the ability of these Affitins to recognize hMSLN on the surface of PM cancer cells (Figure S2B). To do so, we used two PM cell lines: Meso34, from our biocollection of PM cells, which shows negligible MSLN expression, and a Meso34 line that had been transduced to overexpress MSLN (called Meso34-MSLN). Affitins were biotinylated, and binding to Meso34 and Meso34-MSLN cell lines was measured after tetramerization on streptavidin labeled with an A647 fluorophore and quantified by flow cytometry (Figure 2B). Eleven of the 27 Affitin tetramers were able to bind to Meso34-MSLN PM cells with variable binding capacities (Figure 2C). Four of them (named N7, N13, N18, and N23) generated reproducible results and demonstrated superior binding capacities on cells as monomers (Figure S3).

### Characterization of N13 and N18 binding to hMSLN

**Figure 3:**
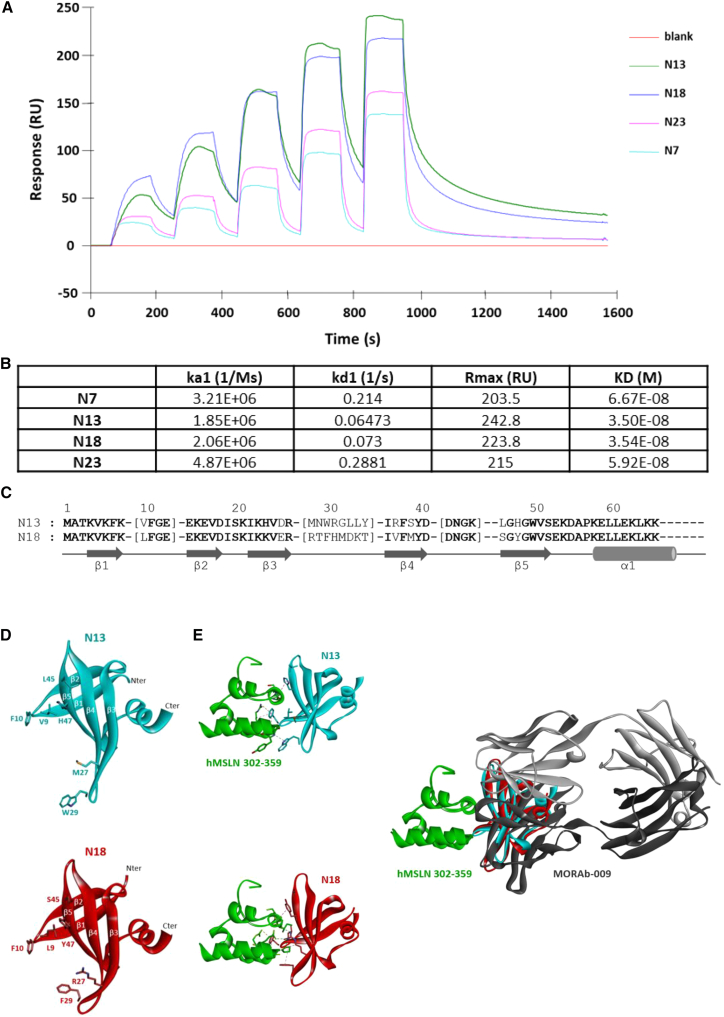
Characterization of the affinity of the most promising antimesothelin Affitins and simulation of their interaction with the hMSLN302-359 mesothelin fragment (A) The binding capacity of N7, N13, N18, and N23 Affitins to the human recombinant mesothelin fragment 302–359 was assessed by surface plasmon resonance and is presented as adjusted sensograms. The affinity constants, calculated using the Langmuir model, are displayed in the table in (B). (C) Sequence alignment of N13 and N18 Affitins. Secondary structure elements are indicated below the sequences. Loops are shown in square brackets, and conserved residues are indicated in bold. (D) Molecular models of N13 and N18 Affitins. Ribbon drawings depict N13 and N18 models composed of five β-strands and one α-helix. The N and C termini are shown, along with some exposed residues predicted to be located in the mesothelin binding site. (E) 3D representation of the Affitin/mesothelin complex. Ribbon drawings of N13 (blue) and N18 (red) Affitins in complex with the mesothelin 302-359 fragment (green). Nonbond interactions are shown as dashed lines. Affitins fully overlap the MORAb-009 binding site (PDB code 4F3F). Images were created using the BIOVIA Discovery Studio visualizer (Dassault Systèmes).

We then proceeded with the characterization of the four promising Affitins identified in the previous experiments. Specifically, we determined their affinity for the recombinant hMSLN302-359 fragment using surface plasmon resonance (SPR) (Figure 3A). All Affitins showed affinities in the nanomolar range, with N13 and N18 displaying the highest affinities of 35.0 nM and 35.4 nM, respectively (Figure 3B). For the N7 and N23 Affitins, dissociation rate constants were higher, resulting in comparatively lower affinities (66.7 nM and 59.2 nM, respectively). We therefore focused on the characterization of the N13 and N18 Affitins. To better understand the interactions between these Affitins and their target, 3D models of N13 and N18 Affitins were generated using AlphaFold3. N13 and N18 Affitins shared 54% sequence identity and 58% sequence similarity between each other (Figure 3C). The two Affitin structural models displayed two β-sheets composed of two (β1β2) and three (β3β4β5) antiparallel β-strands, followed by an amphipathic α-helix. As expected, based on sequence alignment, both models are structurally similar, except for the loop between strands β3 and β4 (Figure 3D). Ribbon representations of N13 and N18 Affitin models show a number of exposed residues expected to be involved in the recognition of hMSLN (Figure 3D). Comparison of the sequences of Affitins able to bind hMSLN (302–359 residues) in flow cytometry (among the 27 initially tested Affitins) with those from the Affitins of the nonbinder group led us to identify some residues potentially implicated in the binding site. These residues (Val/Leu9, Phe10, Met/Arg27, Trp/Phe29, Leu/Ser45, and His/Tyr47 for N13 and N18 Affitins, respectively) are well exposed in both models (Figure 3D). In addition, some residues of the hMSLN fragment could be easily excluded because they were facing the following residues composing the entire hMSLN, according to the hMSLN structure (300–584 residues) extracted from the structure of hMSLN bound to a neutralizing VH antibody (PDB code 8FSL). Both top-ranked MSLN/Affitin complex models generated by AlphaFold3 were obtained with high accuracy, with both interface predicted template modeling (ipTM) and predicted template modeling (pTM) scores >0.8 (ipTM = 0.81 and pTM = 0.85 for N13 Affitin; ipTM = 0.86 and pTM = 0.89 for N18 Affitin). According to the models, most interactions between the two Affitins and hMSLN appear to rely on hydrogen bonds and hydrophobic interactions. These interactions involve the majority of the residues deduced from the comparison of binder and nonbinder groups of Affitins described above. In these models, the Affitins are positioned in the same way at the same recognition site as the MORAb-009 therapeutic antibody (amatuximab), according to the crystallography of the MORAb-009-hMSLN complex (Figure 3E).^24^ To refine our understanding of Affitin binding to MSLN, we tested the ability of N13 Affitin to compete with the MORAB-009 therapeutic antibody for binding to MSLN, using competitive ELISA and SPR. The competitive ELISA results showed a decrease in N13 Affitin binding to the MSLN fragment in the presence of increasing concentrations of MORAb-009 (Figure S4A). The SPR results showed an equilibrium between N13 and MORAB-009 binding signals, with no further increase of the signal after Affitin injection, highlighting competition between the molecules (Figure S4B). This suggests that N13 binds to MSLN in the same area as the MORAb-009 antibody or in a nearby area that causes steric hindrance, consistent with the generated complex models presented in Figure 3E.

Being extremophile Archaea-derived proteins, Affitins are expected to display high thermal stability. We therefore measured the thermal stability of N13 and N18 Affitins under a ramp of temperatures using nanoDSF technology. The N13 Affitin appeared extremely stable, with no thermal denaturation observable up to 95°C (Table 1; Figure S5A). Denaturation was, however, observed around 70°C when a pretreatment with a denaturing agent (1 M guanidium chloride) was performed beforehand, confirming that the apparent stability of N13 measured in native conditions is not an artifact due to the intrinsic sensibility of the technique. The N18 Affitin appeared less stable than N13, with a first thermal denaturation step around 53°C and a second around 81°C (Table 1). Two denaturation steps are rather unusual for such small proteins. These 2 transitions could correspond to an intermediate folding state or to the presence of a dimeric form of Affitin N18. In contrast, the MORAB-009 therapeutic antibody displayed several thermal denaturation transitions, with a first event at 54.5°C, second at 65.2°C, and third at 74.8°C. An aggregation process occurred around 70°C after the second denaturation event (Table 1; Figure S5A). Both molecules displayed good robustness to freeze-thaw cycles (Figure S5B; Table S2).

**Table 1:** Thermal stability values for N13 and N18 monomers and MORAb-009 antibody

Sample ID	Concentration	Initial value		Tm on		Tm1		Tm2		Tm3		Agg on		IP Agg	
		Value	SD	Value	SD	Value	SD	Value	SD	Value	SD	Value	SD	Value	SD
N18 monomer	215 μg/mL	0.09073	0.009	47.21	0.34	53.5	0.03	81.38	0.12	x	x	x	x	x	x
N13 monomer	115 μg/mL	1.2433	0.0006	x	x	x	x	x	x	x	x	x	x	x	x
N13 monomer + 1 M guanidium chloride	115 μg/mL	1.3316	0.001	x	x	68.14	0.45	x	x	x	x	x	x	x	x
N13 dimer	1 mg/mL	1.0828	x	79.9	x	84.4	x	x	x	x	x	79.45	x	83.41	x
MORAb-009	177 μg/mL	0.9763	0.0013	x	x	54.51	0.64	65.23	0.08	74.83	0.7	70.23	0.47	72.16	0.22

Tm, melting point: the temperature at which 50% of the protein is denatured; Tm On, temperature at which denaturation starts; Agg On, onset of aggregation—temperature at which the aggregation process starts; IP Agg, temperature at which 50% of the protein is aggregated.

**Figure 4:**
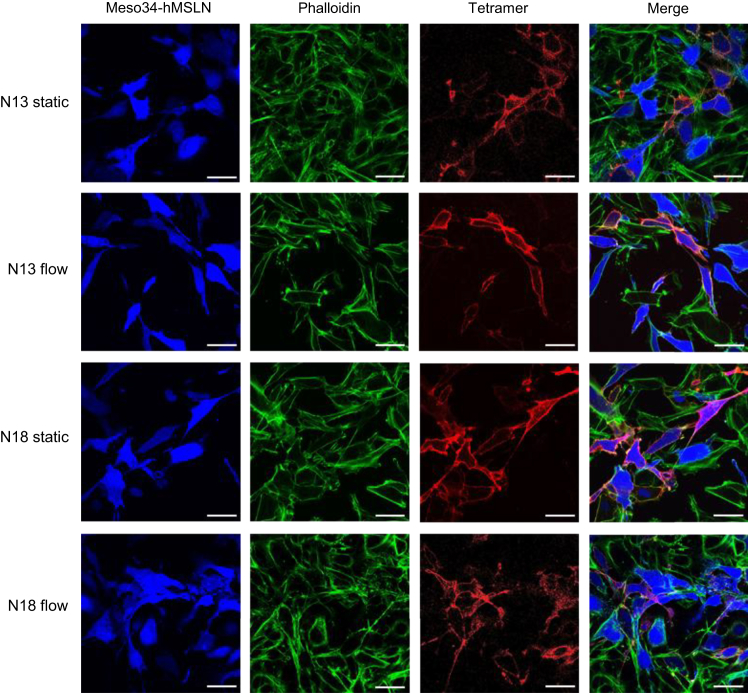
Characterization of the specificity of the most promising antihMSLN Affitins N13 and N18 Affitins were tetramerized on fluorescent streptavidin to facilitate detection. The Affitin tetramers were added to cocultures of fluorescent Meso34-hMSLN (hMSLN+) and unstained Meso34 cells (hMSLN−). Tetramers were either added to the culture medium and incubated statically for 30 min at 37°C or diluted in culture medium and applied to the cocultures under flow (10 μL/min) for 10 min at room temperature. Cells were then washed, stained with phalloidin, and analyzed by confocal microscopy (×60 obj). Scale bars, 20 μm.

To confirm the specificity of N13 and N18 Affitins for hMSLN, we performed coculture experiments of fluorescent Meso34-hMSLN (hMSLN+) PM cells with unstained Meso34 (hMSLN−) PM cells. N13 and N18 fluorescent tetramers were added to the medium of the cocultures either under static conditions or in a flow of culture medium to reproduce hydrodynamic conditions encountered in tumors, particularly in the pleural cavity where PM develops. Pictures of the cocultures acquired by confocal microscopy show specific staining of fluorescent hMSLN+ cancer cells with the two tetramers in both static and dynamic culture conditions (Figure 4). The very low intensity of staining in some blue MSLN+ cancer cells is attributable to the heterogeneity of hMSLN expression within this cell line. Altogether, these results confirm that we isolated at least two lead Affitins able to bind specifically to hMSLN expressed on the surface of PM cancer cells.

### Validation of a homodimer of the N13 Affitin

**Figure 5:**
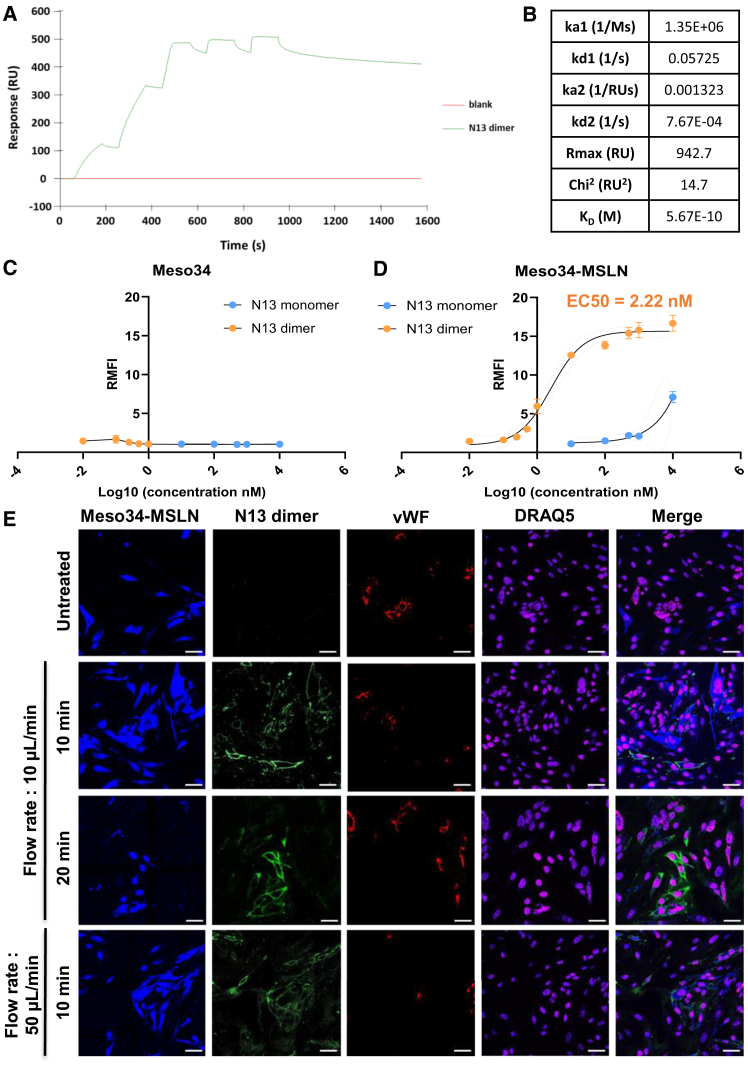
Dimerization of N13 Affitin drastically improves its specific binding to cellular hMSLN (A) A homodimer of the N13 Affitin was produced, and its capacity to bind to recombinant hMSLN fragment 302–359 was assessed by surface plasmon resonance. The affinity constants obtained, calculated with the bivalent model, are displayed in the table in (B). (C–D) Meso34 (MSLN−) and Meso34-MSLN (MSLN+) cells were stained with a concentration range of N13 monomer or dimer, and N13 binding was quantified by flow cytometry (*n* = 3 independent experiments). Results are displayed as the means of ratios of median fluorescence intensities (RMFI) +/− SEM, normalized to the median fluorescence intensity of the secondary antibody alone. (E) N13 dimer was diluted to 1 μM in culture medium, and a flow of this solution was applied multiple times on cocultures of fluorescent Meso34-hMSLN cancer cells (MSLN+), nonfluorescent human fibroblasts, and nonfluorescent human endothelial cells. Nuclei were stained with Draq5, endothelial cells were revealed by von Willebrand Factor (vWF) staining after fixation, and the specificity of dimer binding was assessed by confocal microscopy (×60 obj). Scale bars, 20 μm.

We identified several Affitins able to bind recombinant hMSLN with high affinity. However, we noticed a decrease in affinity—from the nanomolar to the micromolar range—when comparing the binding of Affitins to the recombinant protein with their binding to hMSLN expressed on the surface of cancer cells. This loss of affinity was counteracted by tetramerization of Affitins on fluorescent streptavidin, suggesting that multimerization of Affitins can improve their binding to hMSLN-expressing cells. However, the use of streptavidin is not suitable for clinical applications. We therefore decided to design an Affitin homodimer, consisting of two N13 Affitins linked by a 20-amino-acid, low-immunogenic human muscle aldolase (HMA) linker, previously used to design NK-cell engagers,^25^^,^^26^ to test whether this minimum valency is sufficient to restore efficient binding capacity to cellular hMSLN. Moreover, the dimeric form was retained because of its ease of production and smaller size compared to a tetramer. N13 Affitin was chosen for its higher affinity for cells as a monomer compared to the other candidates and because it appeared to be more stable than the N18 Affitin in our experiments (Table 1). The resulting dimer displayed a molecular weight of approximately 15 kDa, which is still considerably lower than antibodies and equivalent to VHH.^27^ We first assessed the affinity of this construct for the hMSLN302-359 fragment by SPR (Figure 5A). We obtained a very high affinity of the dimer for the recombinant protein, similar to that obtained for the MORAb antibody (Figure S6A), with an apparent K_D_ of 0.57 nM (Figure 5B). Thus, the dimerization of N13 Affitin did not compromise its ability to bind hMSLN302-359 and even enhanced this capability by a factor of 61. While the association rate constant (k_a_) remained largely unchanged between the monomeric and dimeric forms of N13, the enhanced affinity observed for the dimer was primarily driven by an 84-fold reduction in the dissociation rate constant (k_d_). Interestingly, both the k_a_ and k_d_ rate constants are comparable between the bivalent MORAb antibody and the N13 dimer. We then incubated Meso34 (hMSLN−) and Meso34-hMSLN (hMSLN+) cancer cell lines with increasing concentrations of the dimer and analyzed its binding by flow cytometry to determine its affinity for cellular hMSLN. As expected, the dimer was not able to bind to the hMSLN− cell line (Figure 5C). Interestingly, we obtained an approximately 1000-fold increase in the affinity of N13 for cellular hMSLN on the Meso34-hMSLN cell line due to dimerization, with a half-maximal effective concentration (EC_50_) of 2.22 nM (Figure 5D). This affinity is similar to that displayed by the MORAb-009 therapeutic antibody on the same cell line (Figure S6B). To confirm the specificity of the N13 dimer, we applied a flow of culture medium containing the N13 dimer on cocultures of fluorescent Meso34-hMSLN cancer cells, nonfluorescent human fibroblasts (hMSLN−), and nonfluorescent human endothelial cells (hMSLN−). The results show strong, specific binding of the dimer restricted to cancer cells expressing hMSLN, compared to healthy cells in the coculture (Figure 5E). These results demonstrate that the N13 dimer is able to bind cellular MSLN with both high affinity and selectivity in cell cocultures mimicking the tumor microenvironment, even under dynamic culture conditions.

We also compared the thermal stability of the N13 homodimer to that of the monomer using NanoDSF. The dimer appeared to be slightly less thermostable than the monomer, with denaturation observed at 85°C, accompanied by protein aggregation during the process (Table 1; Figure S5A). Nonetheless, this represents a dramatic improvement compared to the thermostability of the MORAB-009 therapeutic antibody. The N13 dimer also displayed good robustness to freeze-thaw cycles (Figure S5B; Table S2).

### Construction and characterization of BiKEs

**Figure 6:**
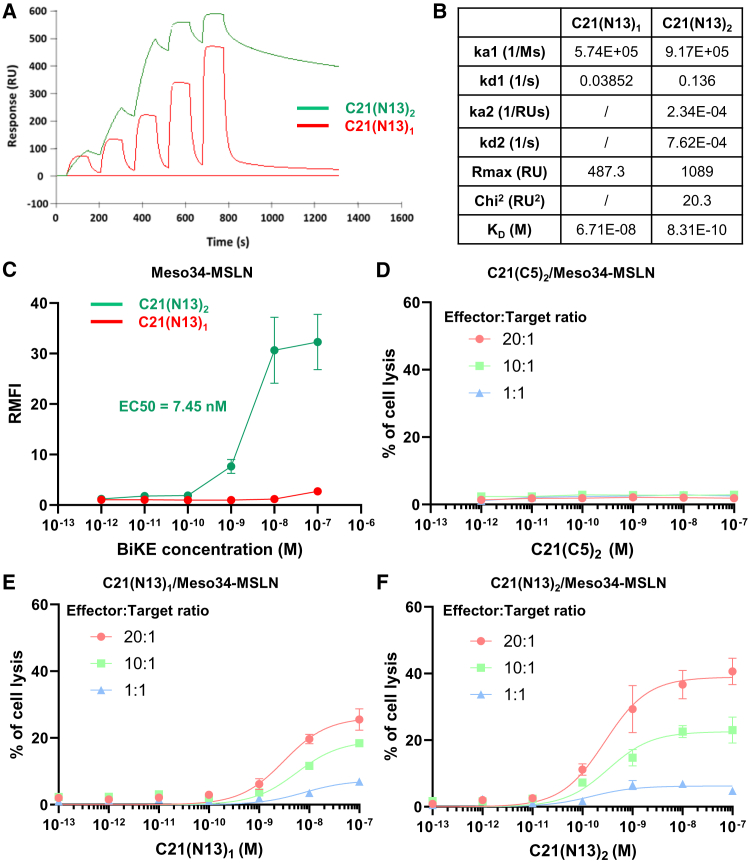
Affitin-based bispecific NK cell engagers efficiently induce NK-mediated cytotoxicity on Meso34-hMSLN cells (A) Bispecific NK engagers (BiKEs), composed of either a monomer (C21(N13)_1_) or a dimer (C21(N13)_2_) of the N13 Affitin fused to the C21 anti-CD16 nanobody, were generated, and their capacity to bind to recombinant hMSLN fragment 302–359 was assessed by surface plasmon resonance. The affinity constants obtained, calculated using the Langmuir model (for C21(N13)_1_) or the bivalent model (for C21(N13)_2_), are displayed in the table in (B). (C) Meso34-hMSLN cells were stained with a concentration range of C21(N13)_1_ and C21(N13)_2_ BiKEs to validate their capacity to bind hMSLN. Binding was quantified by flow cytometry. (D–F) A Cr-51 cytotoxicity assay was performed using the Meso34-hMSLN cell line (hMSLN+) as targets and the NK92CD16h NK cell line as effectors, in the presence of several concentrations of C21(C5)_2_ irrelevant BiKE (D), C21(N13)_1_ (E), and C21(N13)_2_ BiKEs (F). The assay was conducted with effector/target ratios of 20:1 (circles), 10:1 (squares), and 1:1 (triangles). (C–F) Results are expressed as the mean ± SEM of three independent experiments.

Given the attractive targeting properties of the N13 Affitin, we wanted to investigate its potential for therapeutic applications. NK cells are abundant in PM tumors but are usually not effective at killing cancer cells.^28^^,^^29^^,^^30^^,^^31^ We therefore designed BiKEs composed of either a monomer or homodimer of the N13 Affitin fused to the previously described C21 anti-CD16 VHH.^32^ We first evaluated the affinity of the two constructs (respectively named C21(N13)_1_ and C21(N13)_2_) for the recombinant hMSLN302-359 fragment by SPR (Figure 6A). Both constructs were able to bind recombinant hMSLN, with an affinity of 67.1 nM for C21(N13)_1_ and 831 pM for C21(N13)_2_ (Figure 6B). We then validated the capacity of C21(N13)_1_ and C21(N13)_2_ BiKEs to bind both Meso34-hMSLN and NK92CD16h cell lines by flow cytometry, demonstrating the functionality of both components of the BiKEs. As previously observed, the affinity of the C21(N13)_1_ BiKE for Meso34-hMSLN was low (>100 nM), with only slight binding observed at a concentration of 10^−7^ M. The C21(N13)_2_ BiKE showed improved binding capacity due to its dimeric N13, with an EC_50_ of 7.45 nM (Figure 6C). Regarding binding to the NK-92CD16h cell line, C21(N13)_2_ BiKE was able to bind cells from 10^−7^ M, reaching a plateau at 10^−5^ M, suggesting a modest affinity of the construct for CD16 on this cell line (Figure S7). This moderate affinity is, however, desirable to avoid nonspecific activation of NK cells regardless of hMSLN recognition and remains within the range of CD16 affinity for the Fc region of human immunoglobulins (10^−5^ to 10^−7^ M).^33^^,^^34^

Finally, we tested the capacity of the BiKEs to induce NK-mediated cytotoxicity against Meso34-hMSLN cells. We performed a ^51^Cr cytotoxic assay using Meso34-hMSLN cells as targets and the NK92CD16h cell line as effectors. As a negative control to confirm the specificity of the cell lysis, we also performed the experiment with a BiKE composed of the C21 anti-CD16 VHH fused to an irrelevant anti-*S. aureus* Affitin dimer (C21(C5)_2_).^9^ No cytotoxicity was observed with this irrelevant BiKE, as expected (Figure 6D). Both C21(N13)_1_ and C21(N13)_2_ BiKEs induced specific cytotoxicity against Meso34-hMSLN cells, proportional to the effector:target (E:T) ratio (Figures 6E and 6F). NK-mediated cytoxicity was, however, higher with the C21(N13)_2_ BiKE, reaching a maximum of around 40% cell lysis at a 20:1 ratio, compared to around 25% for the C21(N13)_1_ BiKE (Figures 6E and 6F; Table 2). In this BiKE context, we were once again able to observe the gain obtained with Affitin dimerization, as the median effective dose (ED_50_) was improved approximately 11-fold for C21(N13)_2_ compared to C21(N13)_1_ at the 20:1 ratio and 17-fold at the 10:1 ratio (2.77 × 10^−10^ versus 3.11 × 10^−9^ M and 3.25 × 10^−10^ versus 5.62 × 10^−9^ M, respectively) (Table 2).

**Table 2:** Median effective doses and maximal percentages of NK92CD16h-mediated cytotoxicity induced by Affitin-based bispecific NK cell engagers on Meso34-MSLN cells

BIKE	C21(N13)_1_				C21(N13)_2_			
Effector/target ratio	20:1		10:1		20:1		10:1	
	Value (%)	SE	Value (%)	SE	Value (%)	SE	Value (%)	SE
Max cytotoxicity	26.14	1.55	19.18	1.55	38.92	2.10	22.57	1.33
ED50 (M)	3.11E−09	8.89E−10	5.62E−09	1.95E−09	2.77E−10	9.24E−11	3.25E−10	1.17E−10

The median effective dose (ED50) and the maximal cytotoxicity (%) were evaluated for effector-to-target ratios of 20:1 and 10:1 using a “one binding site” nonlinear regression in GraphPad PRISM software.

## Discussion

Targeted therapies against MSLN for cancer treatment have recently elicited growing interest.^35^ In this study, we identified four major Affitins able to bind recombinant hMSLN with high affinities (between 35.0 and 66.7 nM) using ribosome display, NGS, and a cluster-based analysis pipeline. Competition experiments performed with the anti-MSLN therapeutic antibody MORAb-009, along with computational modeling, allowed us to propose a model of the interaction between Affitins and hMSLN. The specificity of Affitins was demonstrated by flow cytometry and confocal microscopy in co-culture models including hMSLN-positive and hMSLN-negative cells, under both static and dynamic culture conditions. A thermal stability evaluation identified an Affitin stable up to 95°C, named N13. Dimerization of this candidate drastically increased its affinity for the recombinant protein (approximately 61-fold) as well as for hMSLN-expressing cells (approximately 1000-fold), without altering its high specificity. Finally, the construction of BiKEs composed of either a monomer or dimer of the N13 Affitin led to efficient NK-mediated specific lysis of hMSLN-expressing PM cells.

All Affitins studied in this work were derived from the Aho7c protein and were part of the L6 library, which was designed to bind targets through both a flat surface and small artificially extended flexible loop. This L6 library is similar to the L4 library, which was designed with the Sac7d scaffold and used in a previous study.^8^ We designed these two kinds of libraries with the aim of enabling Affitins to bind targets through different binding modes. In a previous work aiming to select Affitins against the EpCAM, only the L5 library allowed the generation of binders.^10^ In this study, selections performed with the L5 library, i.e., without the extended loop, were not successful. Thus, depending on the targeted antigen, L5 or L6 libraries may prove more efficient, and this work highlights the importance of using both libraries in parallel for ribosome display selections in future studies.

In our 3D complex models, N13 and N18 Affitins interactions with hMSLN mostly rely on the 27–34 artificially extended loop and, to a lesser extent, on the 45–50 beta-strand—a binding mode previously observed in the crystal structure of the Sac7d Affitin H3-CeldD complex.^8^ We compared the sequences of the 27 selected Affitins to understand differences in their ability to bind hMSLN in ELISA and flow cytometry. We observed that the presence of a cysteine residue induces nonspecific binding in ELISA and prevents any binding on cells. The 9^th^ and 10^th^ amino acid positions appear to be important for Affitins binding capacity, with at least one hydrophobic amino acid in this region required for binding. Overall, a majority of hydrophobic amino acids at positions 9, 10, 27, 29, 45, and 47 appear to favor Affitin binding to cellular hMSLN. The interface between Affitins and hMSLN thus appears to be mostly composed of hydrophobic interactions and hydrogen bonds, as in most protein-protein interactions. No consensus sequence for Affitin could be identified, indicating that the library was sufficiently diverse to offer various independent solutions for binding to hMSLN. Using ELISA and SPR competition experiments, we showed that the N13 monomer and dimer bind hMSLN in the same region as the amatuximab (MORAb-009) therapeutic antibody already used in the clinic. This result validates the 3D complex model of the N13 Affitin proposed in this work (Figure 3E), in which N13 binds the same region of hMSLN as amatuximab.

Unexpectedly, we noticed a strong difference between Affitins affinities on the recombinant protein and on hMSLN-expressing cancer cells. Indeed, we observed approximately 1000-fold lower affinities when Affitins were used as monomers on hMSLN+ PM cancer cells compared to the SPR results. This behavior differs from that observed for the VHH A1, for which an affinity of 15 nM was determined on breast cancer cells,^23^ while we measured an affinity of 15.6 nM by SPR on recombinant hMSLN (Figure S8). This drop in affinity in the cellular model, sometimes observed for VHHs, could be explained by the fact that Affitins recognize their targets largely through a rigid surface, whereas VHHs use the flexible loops of their complementarity-determining regions (CDRs). Consequently, and as observed in a previous study,^36^ Affitins are more sensitive to conformational differences between a recombinant target and the same target presented on the cell surface than VHHs, which can adapt via their CDRs. However, this low affinity of Affitin monomers for cellular targets was overcome in this study by multimerization. We showed that Affitins tetramerized on streptavidin were able to efficiently bind cellular hMSLN with a nanomolar EC_50_ (around 80 nM) and were highly specific for hMSLN even in coculture models including hMSLN+ and hMSLN− cells. This confirms our previous results on the beneficial effect of multimerizing an antibacterial Affitin on dendrimers, which increased affinity by a factor of 600.^37^ We thus designed a homodimer (15 kDa) of Affitin to explore whether this minimal valency would be sufficient to generate a much more efficient molecule. Among the candidates, Affitin N13 demonstrated the most promising properties in terms of affinity and thermal stability. Therefore, we connected two N13 Affitins with a 20-amino acid segment from HMA. We anticipated that the length of this HMA linker would allow the dimer to reach two distant hMSLN molecules on the surface of cancer cells. This dimer displayed a sub-nanomolar affinity (0.57 nM) for the recombinant protein and nanomolar affinity (2.20 nM) for the cellular protein, highlighting a strong avidity effect without any alteration of specificity. Unlike the monomer, the dimer exhibited only a modest reduction in affinity between recombinant and cellular MSLN, with a 4-fold decrease.

It has been extensively documented that low-molecular-weight proteins can distribute more effectively into tissues and penetrate tumors more efficiently than larger molecules such as monoclonal antibodies.^27^^,^^38^ Indeed, with a molecular weight of approximately 15 kDa, the N13 homodimer is smaller than antibody derivatives such as scFvs, while being bivalent and similar to monovalent VHHs. However, low molecular weight is also associated with a reduced serum half-life, which is desirable for imaging applications^39^ but detrimental for therapeutic applications. Other non-Ig scaffolds have therefore been conjugated to polyethylene glycol (PEG) or serum albumin to increase their blood retention, and this strategy has proven efficient in several studies.^3^^,^^40^

As derivatives of extremophile archaea proteins, Affitins often exhibit very high stabilities.^7^^,^^8^^,^^41^ We demonstrated that the N13 monomer and N13 homodimer maintain their organized structure up to 95°C and 85°C, respectively, whereas amatuximab (MORAb-009) exhibited thermal denaturation as early as 54°C, followed by aggregation around 70°C (Table 1; Figure S5). N13 and its dimer were also stable after repeated freeze-thaw cycles. This property of Affitins is noteworthy, as low thermal stability is often associated with protein aggregation, a problem particularly observed and studied for antibodies.^42^ Affitins are small, nonglycosylated proteins without disulfide bonds that can be efficiently produced in *E. coli* using standard bacterial expression systems. Their production relies on inexpensive media and simple purification schemes, such as affinity chromatography (e.g., His-tag/Ni-NTA), typically yielding tens to hundreds of milligrams of purified protein per liter of culture. In contrast, mAbs require eukaryotic expression systems (e.g., CHO or HEK293 cells) to ensure proper folding and glycosylation. These systems involve costly culture media, longer production times, and more complex downstream processing, primarily using Protein A affinity chromatography, all of which substantially increase the overall production cost. A comparative analysis by Lebozec et al. evaluated the production of a Fab fragment in *E. coli*, *Pichia pastoris*, and CHO cells.^43^ The CHO platform was by far the most expensive, both in terms of capital investment and consumables. When all direct and indirect costs were considered, the cost of goods (CoG) for CHO-based production remained the highest. While precise CoG values depend on production scale, process optimization, and the protein of interest, it is reasonable to expect that Affitins—being smaller and structurally simpler than Fab fragments and monoclonal antibodies—would exhibit even lower CoG when produced in bacterial systems. However, Affitins can also be produced in HEK and CHO cells, making them compatible with the manufacturing pipelines of private pharmaceutical companies.

As the N13 Affitin contains no cysteine residue, the insertion of a cysteine at its C-terminus would enable simple site-specific conjugation of a wide panel of molecules of interest, such as chemotherapies or nanovectors, as we have previously demonstrated.^37^^,^^44^ Furthermore, the high thermic stability of N13 would be compatible with metal chelation, a process that typically requires high temperatures.

The intrinsic properties of non-Ig scaffolds can be exploited for specific applications in cancer therapy.^3^^,^^4^^,^^45^ Moreover, while multivalency is relatively difficult to achieve with monoclonal antibodies, non-Ig scaffolds can more easily be fused to other targeting moieties to generate bi- or multispecific constructs.^45^ One main criterion for choosing Sul7d proteins as a scaffold was that their N and C termini are distant from the binding site, enabling fusion to other proteins at both ends. An example of fusing two Affitins was previously proposed to target epidermal growth factor receptor (EGFR) and programmed cell death protein 1 (PD-1) simultaneously.^46^ We have also designed several Affitin fusion proteins with, for instance, a green fluorescent protein or an alkaline phosphatase and demonstrated that both Affitins and their partners remained functional once linked together.^6^^,^^9^ Another application for such small proteins would be the functionalization of nanovectors, a field in which steric hindrance is a critical parameter^13^^,^^37^

To evaluate the therapeutic potential of anti-MSLN Affitins, we designed BiKEs composed of either an Affitin monomer or dimer fused to an anti-CD16 VHH to activate natural killer cells toward hMSLN-expressing cancer cells. We observed a maximum of 40% of cytotoxicity with the N13 dimeric BiKE at the 20:1 ratio against the Meso34-hMSLN cell line. Again, results obtained with the monomeric and dimeric Affitins in BiKE constructs highlighted the benefit of dimerization, as the effective dose decreased by a factor of 11 with the dimeric BiKE. Del Bano et al. studied a Fab-like bispecific format (bsFab) designed with the anti-CD16 VHH C21 and anti-hMSLN VHH A1 fused to human CH1 and Ck IgG domains as a heterodimerization motif.^47^ After 12 h of co-incubation, this bsFab NK engager induced NK cell-mediated specific cytotoxicity, with maximal lysis of 77% and 35% for the HCC1806 and MDA-MB-231 cell lines, respectively, and EC_50_ values ranging from 0.026 to 0.919 nM (E:T ratio = 10:1). In another study, a high-affinity tri-specific killer engager was designed by fusing an anti-hMSLN scFv and the anti-CD16 VHH cam16, linked together by IL-15 and HMA peptidic linkers.^48^ Thirty nanomolars of this engager was shown to kill two hMSLN-expressing cell lines (A549 and H460) at levels of approximately 80% and 100%, respectively (E:T ratio = 10:1) after 6 h of coincubation. These studies used either a longer coincubation time with NK cells, different target cell lines, or higher concentrations of natural killer cell engagers (NKCEs), without providing information about EC_50_, which makes comparison difficult. Meso34 is a mesothelioma cell line established from pleural effusions (PEs) of patients in our laboratory and transfected to express high levels of hMSLN on its surface.^49^ However, the range of cell lysis obtained with the BiKE is consistent with that observed with specific CD8 T cells on PM cells, which ranges from 20% to 60% depending on the PM cell line used.^50^ Further work is needed to investigate whether an intrinsic characteristic of Meso34-hMSLN cells could explain why a fraction of these cells are resistant to lysis induced by C21-(N13)_2_. Overall, our results compare favorably with other anti-hMSLN NKCEs in different cancers, both in terms of efficiency and potency, with variability depending on the cell line studied.^47^^,^^48^

Anti-hMSLN Affitin BiKEs thus illustrate one of the many potential applications of Affitins in cancer therapy. In PM, it has been shown that tumors are infiltrated by NK cells.^28^^,^^29^^,^^30^^,^^31^^,^^51^ In particular, high NK cell infiltration in patients with epithelioid PM is associated with significantly better overall survival.^51^ This suggests that activation of NK cells could be a promising immunotherapeutic strategy for the treatment of epithelioid PM. The use of BiKEs therefore appears attractive for triggering the reactivation of NK cells against PM tumors.

## Material and methods

### Protein production

All synthetic DNA sequences coding for proteins were supplied by GeneCust. The sequences of the proteins produced for this study are shown in Table S1. All proteins, except VHH A1, were expressed in the cytoplasm of *E. coli*. After purification, the homogeneity and size of the proteins were evaluated by sodium dodecyl sulfate-polyacrylamide gel electrophoresis (SDS-PAGE) on a 15% polyacrylamide gel. Protein concentrations were determined by measuring the optical density at 280 nm (OD 280) of the protein solutions (molar extinction coefficients of proteins are indicated in Table S1). All proteins were aliquoted and stored in PBS, pH 7.4, at −80°C. Protein sequences are described in Table S1.

#### Production of the biotinylated N-terminal fragment of MSLN

The synthetic sequence corresponding to the N-terminal 302–359 fragment of hMSLN was inserted into the pFP1312 plasmid via BamHI and HindIII restriction sites. This plasmid, derived from the pQe30 vector (QIAGEN), enables the expression of proteins fused to an AviTag and a hexahistidine tag at their C terminus. This plasmid was used to transform the *E. coli* Origami B strain (Merck) containing the pBirA plasmid (Avidity), and hMSLN 302–359 was produced as previously described with some modifications.^24^ Briefly, 1 L of 2YT medium (Euromedex) containing 100 μg/mL ampicillin (Euromedex), 25 μg/mL kanamycin (Euromedex), 50 μg/mL tetracycline (Euromedex), 50 μM biotin (Thermo Fisher Scientific), and 0.1% glucose (Euromedex) was inoculated with 20 mL of an overnight starter culture. The culture was grown at 37°C to an OD_600_ of 0.8, and isopropyl β-D-1-thiogalactopyranoside was added to a final concentration of 0.5 mM. After 16 h of incubation at 30°C, bacteria were harvested by centrifugation and lysed using an Emulsiflex C3 (Avestin) in buffer A, containing 25 mM Tris, pH 7.5, 150 mM NaCl, 10% glycerol, 20 mM imidazole (reagents from Euromedex), 5 μg/mL DNAse I (Thermo Fisher Scientific), and 1 tablet of EDTA-free protease inhibitor cocktail (Roche). The bacterial lysate was centrifuged at 12 000 g for 30 min, and the supernatant was loaded onto a 1 mL column packed with Ni-NTA resin (Cytiva) equilibrated with buffer A to capture the His-tagged hMSLN 302–359. The column was washed with buffer A, and the protein was eluted with buffer A containing 250 mM imidazole. The protein was further purified by size-exclusion chromatography on a Superdex 75 gel filtration column (Cytiva) using PBS, pH 7.4, as a running buffer.

#### Production of Affitins

The nucleotide sequences of Affitins were synthesized and inserted via BamHI and HindIII restriction sites into the pFP1001 plasmid,^52^ a derivative of the pQe30 vector (Qiagen) encoding proteins fused to an RGS-hexa-histidine tag at their N terminus. The Affitins were also sub-cloned into the pFP1301 plasmid via BamHI and HindIII restriction sites. The pFP1301 plasmid is derived from the pQe30 vector (Qiagen) and expresses proteins fused with an RGS-hexa histidine tag at their N-terminal and an AviTag at their C-terminal to allow *in vivo* biotinylation. The homodimeric version of the N13 Affitin was built by connecting two N13 Affitins with a linker encoding a 20-amino-acid segment of HMA.^25^ The resulting sequence was inserted into the pFP1301 plasmid via BamHI and HindIII restriction sites. These plasmids were used to transform the *E. coli* DH5α I^q^ strain (Thermo Fisher Scientific), containing the plasmid pBirA when biotinylation was desired. The Affitins were expressed and purified as described previously^10^ using a Ni-NTA resin (Qiagen) and size-exclusion chromatography on a Superdex 75 gel filtration column (Cytiva) equilibrated with PBS, pH 7.4.

#### Production of the biotinylated BiKE

BiKEs were built by genetically fusing the anti-CD16 C21^53^ VHH to either a monomeric (C21(N13)_1_) or a homodimeric (C21(N13)_2_) N13 Affitin using a linker derived from the HMA sequence.^25^ An irrelevant BiKE composed of a homodimer of the C5 Affitin, specific for *S. aureus*^9^, fused to the C21 VHH via the HMA linker, was also produced and used as a negative control in the experiments. The nucleotide sequences of the monomeric and dimeric N13 Affitin, as well as the dimeric C5 Affitin, were inserted via BamHI and HindIII restriction sites into the pFP1601 vector, which encodes the C21-HMA at the N-terminal of the Affitin and an RGS-hexa-histidine tag followed by an AviTag at their C-terminal. These plasmids were used to transform the *E. coli* SHuffle Express strain (New England Biolabs) containing the pBirA plasmid, and the BiKEs were expressed and purified as described for Affitins.

### Selection of hMSLN-specific binders by ribosome display

The biotinylated extracellular domain of recombinant hMSLN (R&D Systems) and the biotinylated hMSLN 302–359 fragment, produced as described above, were used for the selection of Affitins by ribosome display. These two proteins were used alternatively from round to round to drive the selection toward the region of hMSLN recognized by both the monoclonal antibody MORAb-009^24^ and the VHH A1.^23^ The selection scheme is shown in Figure S1. A detailed procedure is provided in the supplemental information.

### NGS

To prepare samples for NGS, each pool of DNA sequences obtained after selection was treated as follows. A PCR was performed using adapter primers (Eurofins) NGS_RDV2_H5_Fint (5′-TCGTCGGCAGCGTCAGATGTGTATAAGAGACAGATCCGCGACCAAAGTAAAATTC-3′) and NGS_RDV2_H5_Rint (5′-GTCTCGTGGGCTCGGAGATGTGTATAAGAGACAGAGCTTCAGTTTCTCCAGCAG-3′), 5 ng of DNA, and 1 U of Phusion polymerase (Thermo Fisher Scientific) in the presence of 4% DMSO (Thermo Fisher Scientific). The PCR program consisted of an initial denaturation at 98°C for 30 s, followed by 20 cycles of 98°C for 10 s, 66°C for 30 s, and 72°C for 10 s, with a final elongation step at 72°C for 5 min. The amplified DNA was purified using the Promega Wizard SV Gel and PCR Clean-up kit, and the concentration of the purified PCR product was estimated by UV absorbance measurement. The homogeneity of the products was controlled on a 1.5% agarose gel. A second PCR was then performed to add Nextera adapters (Illumina). Amplicons were validated on a DNA1000 bioanalyzer (Agilent), and quantification was performed by qPCR. Finally, amplicons were sequenced on a MiSeq 300-cycle Micro v2 (PE150), obtaining on average 1 million paired-end reads per sample.

Cluster analysis was performed after translation of the DNA sequences obtained from the different selection rounds into proteins and is described in detail in the supplemental information. All bioinformatic analyses were performed using Python 3.8.5 and standard scientific packages. Logo representations were generated using the Logomaker package.^54^ The source code with a graphic interface is available on GitHub: https://github.com/TacienP/BPSA. For practical purposes, we arbitrarily selected 25 Affitin sequences (N1 to N25) representative of the diversity of clusters for protein production and *in vitro* characterization. For comparative experiments, 2 additional Affitins identified by ELISA were included, resulting in a total of 27 Affitins to be evaluated.

### Cell culture

The Meso34 PM cell line is part of a validated biocollection (Ministère de l’Enseignement Supérieur et de la Recherche n° DC-2017-2987 and Commission Nationale de l’Informatique et des Libertés [CNIL] n° 1657097) and was approved by the local independent ethical committee (CPP Ouest-IV-Nantes). This cell line was established from the PE of a patient in our laboratory.^55^ PE from patients with suspected mesothelioma was aseptically collected by thoracocentesis at Laennec Hospital (St-Herblain, France). Diagnoses were established through both fluid cytology and immunohistochemical staining of pleural biopsies performed by the pathology department at Laennec Hospital and externally confirmed by the French panel of pathology experts for the diagnosis of mesothelioma (Mesopath). Samples were collected in accordance with the standards set forth in the Declaration of Helsinki. All recruited patients had received no prior anticancer therapy and provided signed informed consent. The stable Meso34-MSLN cell line was obtained by transduction of Meso34 cells with an hMSLN-encoding lentivirus (a gift from the Adusumilli PS lab, MSK, New York, USA). MSLN-overexpressing Meso34 cells were then sorted by flow cytometry and expanded for the experiments. Meso34-MSLN cells were further transduced with another lentivirus to enable stable cytoplasmic expression of blue fluorescent protein (BFP) for the coculture experiments (Addgene). The resulting cell line is referred to as Meso34-MSLN/BFP. The NK-92 cell line from the American Type Culture Collection (ATCC) (CRL-2407) was stably transfected with a vector encoding human CD16 (NK92CD16h) and used as the effector in these experiments (gift from Béatrice Clémenceau).^56^ All PM cell lines and the NK92CD16h cell line were maintained in RPMI-1640 medium (Gibco) supplemented with 2 mM L-glutamine, 100 IU/mL penicillin, 0.1 mg/mL streptomycin, and 10% heat-inactivated fetal calf serum (Gibco). NK92CD16h cells were diluted to 0.1 × 10^6^ cells/mL and stimulated with 200 U/mL of IL-2 (Proleukin, Chiron) every 3 to 4 days. Human foreskin fibroblasts-2 (HFF-2) were purchased from ATCC and cultured in DMEM medium (Gibco) supplemented with 2 mM L-glutamine, 100 IU/mL penicillin, 0.1 mg/mL streptomycin, and 10% heat-inactivated fetal calf serum (Gibco). Primary human umbilical vein endothelial cells (HUVEC) were isolated in the laboratory from human umbilical cords and maintained in endothelial cell growth medium-2 (EGM-2) medium (Promocell) supplemented with 0.02 mL/mL fetal calf serum, 5 ng/mL epidermal growth factor, 10 ng/mL basic fibroblast growth factor, 20 ng/mL insulin-like growth factor, 0.5 ng/mL vascular endothelial growth factor, 1 μg/mL ascorbic acid, 22.5 μg/mL heparin, and 0.2 μg/mL hydrocortisone (all from Promocell). All cells were cultured at 37°C in a 5% CO_2_ atmosphere.

### ELISA

Maxisorp plates (Thermo Fisher Scientific) were coated with either 100 μL of 2 μg/mL recombinant hMSLN (R&D Systems, 3265-MS) or 66 μM NeutrAvidin (Thermo Fisher Scientific) and blocked with 300 μL of PBS containing 0.5% bovine serum albumin (BSA) (Sigma-Aldrich). The AviTag-biotinylated recombinant human 302–359 MSLN fragment was added to each well of the NeutrAvidin-coated plate (150 nM, 100 μL/well), an amount sufficient to saturate all biotin-binding sites. For the BSA control plate, the Maxisorp wells were coated only with 100 μL of PBS containing 0.5% BSA. After a blocking step with PBS containing 0.5% BSA, 100 μL of purified AviTag-biotinylated Affitins were added to the wells at a final concentration of 1 μM, and detection was performed with a horseradish peroxidase (HRP)-streptavidin conjugate (Thermo Fisher Scientific). OD at 450 nm was measured on a spectrophotometer. All steps were performed at room temperature with 1 h incubation in 100 μL PBS pH 7.4, and washes were performed with PBS pH 7.4 containing 0.1% Tween 20 (Thermo Fisher Scientific).

### Flow cytometry

All staining procedures were performed in PBS containing 0.1% BSA (Sigma-Aldrich).

#### Screening of Affitin-streptavidin complexes

Biotinylated Affitins were tetramerized on streptavidin coupled to Alexa Fluor 647 (streptavidin-AF647) (Thermo Fisher Scientific, S21374) by incubating the fluorescent streptavidin with a 50-fold molar excess of each biotinylated Affitin for 30 min at room temperature under shaking. Meso34 and Meso34-MSLN cells were stained in suspension with 80 nM of the Affitin tetramers on streptavidin-AF647 or with streptavidin AF-647 alone for 30 min at 4°C. Cells were then washed twice and analyzed on a BD FACSCanto II (Becton Dickinson, Grenoble, France).

#### N13 monomer, N13 dimer, and MORAb-009 staining of Meso34 and Meso34-MSLN cells

Meso34 and Meso34-MSLN cells were stained in suspension with a concentration range of N13 dimer or N13 monomer for 30 min at 4°C. Cells were washed twice with PBS, and the dimer was revealed by incubation with 1 μg/mL of an anti-6X His Tag-PE antibody (Abcam, ab72467) for 30 min at 4°C. The monomer was revealed with 4 μg/mL streptavidin-PE (Thermo Fisher Scientific, 12-4317-87) for 30 min at 4°C. For staining with MORAb-009, Meso34-MSLN cells were stained in suspension with a concentration range of the MORAb-009 antibody (Proteogenix, PX-TA1049) for 30 min at 4°C. Cells were washed twice with PBS, and the MORAb-009 was revealed by incubation with 1 μg/mL of Dylight 488-conjugated goat antihuman IgG Fc antibody (Thermo Fisher Scientific, SA5-10134) for 30 min at 4°C. For all conditions, cells were washed twice again with PBS and analyzed on a BD FACSCanto II. Data were analyzed using FlowJo v.10 software (BD Biosciences). The EC_50_ was calculated with GraphPad PRISM software using a nonlinear “one binding site” regression.

#### Validation of NK cell engager binding

Meso34-MSLN and NK92CD16h cells were incubated with increasing concentrations of C21(N13)_1_ and C21(N13)_2_ BiKEs for 30 min at 4°C. Cells were washed, and BiKEs were revealed by incubation with 4 μg/mL streptavidin-PE (Thermo Fisher Scientific, 12-4317-87) for 30 min at 4°C. Cells were then washed and analyzed on a BDFACS Canto II flow cytometer (Becton Dickinson, Grenoble, France). BiKEs were calculated using GraphPad PRISM software with a nonlinear “one binding site” regression.

### SPR: Kinetics analysis

SPR experiments were performed by injecting a series of analyte concentrations over an immobilized MSLN fragment. The biosensor used in this study was a Biacore T200 instrument (GE Healthcare). CM5 research-grade sensor chips (carboxymethyl-dextran surface) and HBS-EP running buffer (0.01 M HEPES, pH 7.4; 0.15 M NaCl; 0.005% [v/v] surfactant P20; 3 mM EDTA) were also purchased from GE Healthcare. Recombinant MSLN fragment (hMSLN 302–359) was coupled at approximately 700 RU on the carboxymethyl-dextran surface of a CM5 chip following the standard amine-coupling protocol. N7, N13, N18, and N23 Affitins; the N13 dimer; and C21(N13)_1_ and C21(N13)_2_ BiKEs were diluted in HBS-EP buffer at concentrations ranging from 12.5 to 200 nM and injected on the MSLN-coated chip in single cycle kinetics (SCK) mode. The flow rate was set up at 30 μL/min, and association and dissociation were allowed for 2 and 10 min, respectively. A 10 mM NaOH solution was injected over the chip for 30 s to regenerate between each cycle. Rmax (RU), *k*_on_ (M^−1^·s^−1^), *k*_off_ (s^−1^), and *K*_D_s (M) were calculated from kinetic sensorgrams using the Langmuir model (for Affitin monomers, VHH A1, and the C21(N13)_1_ BiKE) or the bivalent model (for MORAb-009, the N13 dimer, and the C21(N13)_2_ BiKE), adapted for SCK analysis with the Biacore T200 Biaevaluation Software 3.1.

### Confocal microscopy

#### N13 and N18 tetramers

Affitins were tetramerized on A647-streptavidin (Thermo Fisher Scientific, S21374) by incubating the fluorescent streptavidin with a 50-fold molar excess of each monomeric Affitin for 30 min at room temperature with shaking. Meso34-MSLN cells also expressing BFP (Meso34-MSLN/BFP) were seeded with nonfluorescent Meso34 cells at a 50:50 ratio in 6-channel μ-Slide VI 0.4 IbidiTreat flow slides, with 25,000 total cells per channel in 200 μL of the appropriate medium, and incubated overnight at 37°C in a 5% CO_2_ atmosphere. The next day, a 20 nM solution of N13 or N18 fluorescent Affitin-streptavidin complexes, diluted in complete RPMI medium, was either added to the cultures under static conditions or under a flow of 10 μL/min for 10 min at room temperature. Cells were then washed twice with PBS, fixed with 4% paraformaldehyde (Thermo Fisher Scientific) for 20 min, stained with 5 U/mL A488-phalloidin (Invitrogen A12379) in PBS-BSA 1%-Triton 0.5% for 10 min at room temperature, and observed on a confocal SIM microscope using a 60× objective.

#### N13 dimer

Meso34-MSLN/BFP cells were seeded with nonfluorescent HFF-2 and HUVEC cells at a 40:30:30 ratio in 6-channel μ-Slide VI 0.4 IbidiTreat flow slides, with 35,000 total cells per channel in 200 μL of HUVEC culture medium, and incubated overnight at 37°C in a 5% CO_2_ atmosphere. The next day, the cultures were incubated with a 1 μM solution of N13 dimer diluted in HUVEC medium for 10 or 20 min at room temperature under a flow rate of 10 μL/min or 20 μL/min. Cells were then washed twice with PBS, fixed with 4% paraformaldehyde (Thermo Fisher Scientific) for 20 min, and the dimer was revealed by incubation with 2.5 μg/mL mouse anti-AviTag antibody (GenScript, A01738) followed by 2 μg/mL donkey anti-mouse-A488 antibody (Invitrogen A21202) for 30 min at 4°C each. Endothelial cells were stained with 1 μg/mL anti-Von Willebrand Factor antibody (Abcam, ab6994) and 2 μg/mL goat anti-rabbit Alexa Fluor 594 antibody (Molecular Probes, 8889S) for 30 min at 4°C. DRAQ5 (Thermo Fisher Scientific, 62251) was added at 5 μM for 5 min at room temperature before the cells were finally washed twice and observed on a confocal Nikon SIM microscope using a 60× objective.

### Structure prediction

hMSLN/Affitin complexes were generated for both N13 and N18 Affitins using AlphaFold3. AlphaFold3 was accessed through its public web server (https://alphafoldserver.com). The amino acid sequence of MSLN (residues 302–359 in the Uniprot sequence Q13421), together with those of either N13 or N18 Affitin, was input into the server, and AlphaFold3 generated five models per complex, ranked by the AlphaFold3 ranking score. Confidence in the predicted complex was assessed using pTM (predicted template modeling) and ipTM (interface predicted template modeling) scores. Both ipTM and pTM scores >0.8 indicated highly confident, high-quality predictions.^57^

### NK cell cytotoxicity assays

Meso34-MSLN cells were used as targets in this experiment. The cells were first incubated for 1 h with 50 μCi of ^51^Cr (PerkinElmer) at 37°C to enable labeling and then washed five times to remove free ^51^Cr. Labeled Meso34-MSLN cells were resuspended in culture medium at a concentration of 0.09 × 10^6^ cells/mL, and 33 μL/well (corresponding to 3,000 cells) were plated in a 96-well V-bottom plate. Thirty-three microliters of a suspension of NK92CD16h effector cells were added to each well at E:T ratios of 20:1, 10:1, and 1:1. 33 μL of serial dilutions (ranging from 10^−7^ M to 10^−13^ M) of C21(N13)_1_, C21(N13)_2_, and C21(C5)_2_ BiKEs were added to the corresponding wells. For the controls, the spontaneous release of ^51^Cr by ^51^Cr-radiolabeled Meso34-MSLN target cells was evaluated by incubating 3,000 cells in culture medium alone. Maximum ^51^Cr release was determined by adding 1% Triton X-100 to 3,000 labeled Meso34-MSLN cells. Each condition of the assay was tested in triplicate. Plates were incubated for 4 h at 37°C, centrifuged, and 25 μL of supernatant from each well was transferred into a flexible 96-well plate containing 100 μL of Ultima Gold XR scintillation liquid (PerkinElmer) per well. Plates were sealed with a plastic cover sheet, and the quantity of ^51^Cr released during the assay was quantified using a 1450 MICROBETA Jet beta counter (Wallace). The percentage of specific lysis was expressed as Specific lysis (%) = ([experimental release − spontaneous release]/[maximal release − spontaneous release]) × 100. The median effective dose (ED50) was calculated using GraphPad PRISM software (nonlinear “one binding site” regression).

## Data and code availability

The datasets used and/or analyzed during the current study are available from the corresponding authors upon reasonable request. The source code with a graphic interface is available on GitHub: https://github.com/TacienP/BPSA.

## Acknowledgments

The authors thank INSERM, CNRS, Région Pays de la Loire (Research program: “Paris Scientifiques”), the Ligue contre le cancer Grand-Ouest (Committee CD17, CD22, and CD44), the Ligue contre le cancer nationale (Tina Briolay grant), and Inserm Transfert for their financial support. The authors also thank the cluster LUNG innOvatiOn (LUNG O2) for logistic support; the National Research Agency under the Programme d'Investissements d'Avenir (ANR-16-IDEX-0007); the Cytometry Facility “CytoCell” from Nantes. We acknowledge the MicroPICell core facility (SFR Bonamy, BioCore, Inserm UMS 016, CNRS UAR 3556, Nantes, France), member of the Scientific Interest Group (GIS) Biogenouest, IBISA, and the national infrastructure France-Bioimaging supported by the French national research agency' (ANR-24-INBS-0005 FBI BIOGEN). This work was partly supported by state aid managed by the National Research Agency under the France 2030 program, referencing ACCREDIA ANR-22-PEBI-0009. The authors are particularly grateful to the IBiSA Recombinant Protein Facility (P2R, Inserm BioCore US16, SFR Bonamy, Nantes), supported by funding from IBiSA, the Biogenouest network, and the Région Pays de la Loire, as well as the Imp@ct core facility (SFR Bonamy, Nantes Université, CNRS, Inserm, Nantes, France), a member of the Scientific Interest Group Biogenouest, for technical support. The authors also thank Pr. Prasad S. Adusumili for providing the human MSLN-encoding lentivirus and Dr. Béatrice Clémenceau for providing NK92CD16h cells. Finally, the authors thank Lucas Treps for the graphical abstract (created in BioRender. Treps, L. [2025] https://BioRender.com/j78n447).

## Author contributions

T.B. performed the experiments and wrote the manuscript. T.P. conducted the NGS sequencing analysis, selected the Affitin sequences studied in the project, and reviewed the manuscript. J.F. performed the microscopy experiments. T.B. and S.L. conducted the cellular experiments. H.G. carried out the ribosome display selections. A.Q. and E.M. performed the Affitin modeling. M.M. performed and analyzed all SPR experiments. P.C. carried out the competition ELISA and protein production. K.B. and A.F. performed and analyzed NanoSDF experiments. F.D. and F.P. designed the BiKEs, and F.D. tested them. F.P. produced the BiKEs and Affitins. C.B. and F.P. designed and supervised the project. F.P., C.B., N.B., B.M., and F.D. revised the manuscript. All authors approved the final version of the manuscript.

## Declaration of interests

F.P. is the inventor of a patent application (PCT/IB2007/004388), owned by the Institut Pasteur and the Center National de la Recherche Scientifique (CNRS), which covers a process for the generation of Affitins. F.P. is a co-founder of a spin-off company of the Institut Pasteur/CNRS/Université de Nantes, which holds a license agreement related to this patent application. T.B., T.P., F.D., F.P., and C.B. are inventors of a patent on antihMSLN Affitins (PCT/EP2024/087379).

## Supplemental information

Document S1. Figures S1–S8 and Tables S1 and S2

Document S2. Article plus supplemental information
