# Supplementary material for: Development of potent Affitin-based bispecific NK cell engagers for the therapy of MSLN-expressing cancers
Source: Mol Ther Oncol. 2025 Nov 19;33(4):201095. doi: 10.1016/j.omton.2025.201095 (PMC12752793; doi:10.1016/j.omton.2025.201095)
Supplement: Document S1. Figures S1–S8 and Tables S1 and S2 [file mmc1.pdf]

OMTON, Volume *33*

Supplemental information

Development of potent Affitin-based bispecific NK cell engagers for the therapy of MSLN-expressing cancers

Tina Briolay, Tacien Petithomme, Hermelyne Gravoueille, Judith Fresquet, Sylvia Lambot, Pauline Cossard, Barbara Mouratou, Agnès Fortun, Karine Bernardeau, Agnès Quéméner, Mike Maillasson, Nicolas Boisgerault, Erwan Mortier, François Davodeau, Frédéric Pecorari, and Christophe Blanquart

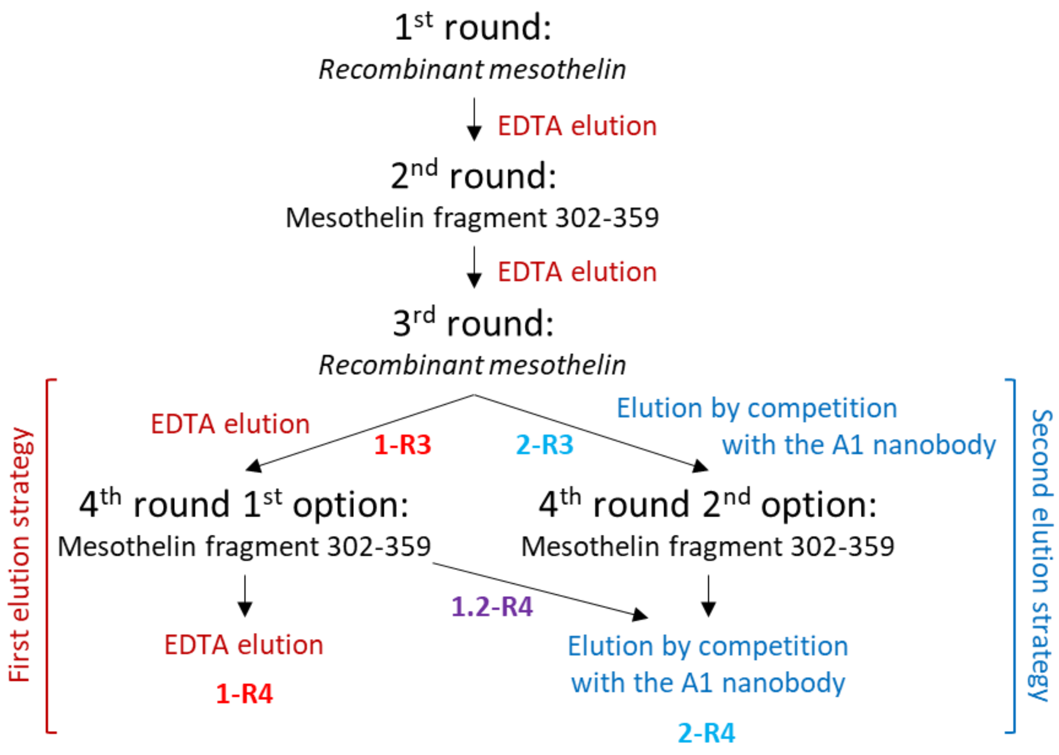

Figure S1: Ribosome display selection strategies used for anti-MSLN Affitins selection. Schematic representation of the ribosome display selection strategies used in this study.

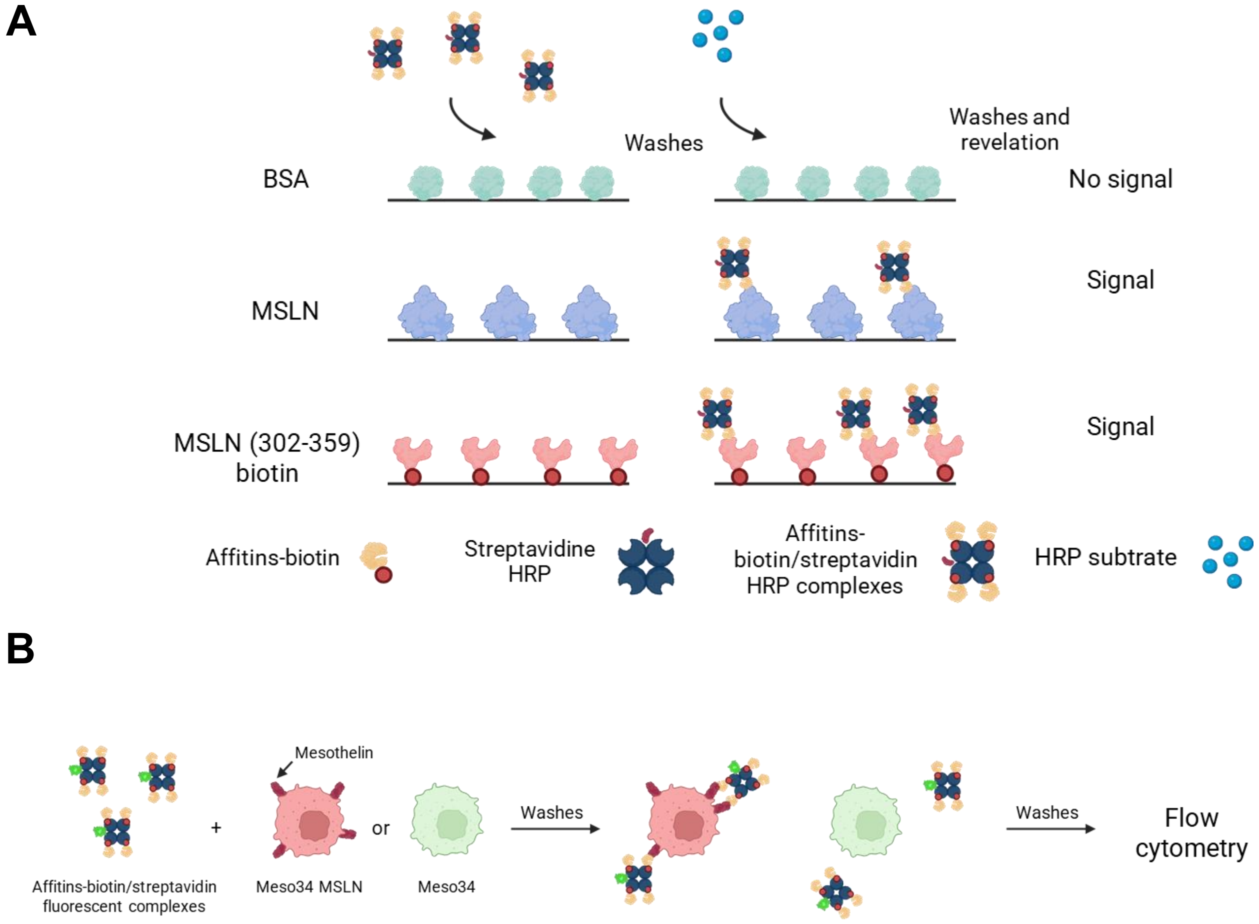

Figure S2: Schemes representing methodologies used to identify Affitins specific for Mesothelin (MSLN). A) Scheme of the ELISA assay strategy used. BSA was used as a negative control to identify nonspecific Affitins. MSLN was used to identify Affitins recognizing our target of interest. MSLN (302-359) was used to identify Affitins binding on the epitope recognized by anti-MSLN antibody MORAb-009 (Amatuximab). B) scheme of the methodology used to evaluate recognition of cellular MSLN by Affitins.

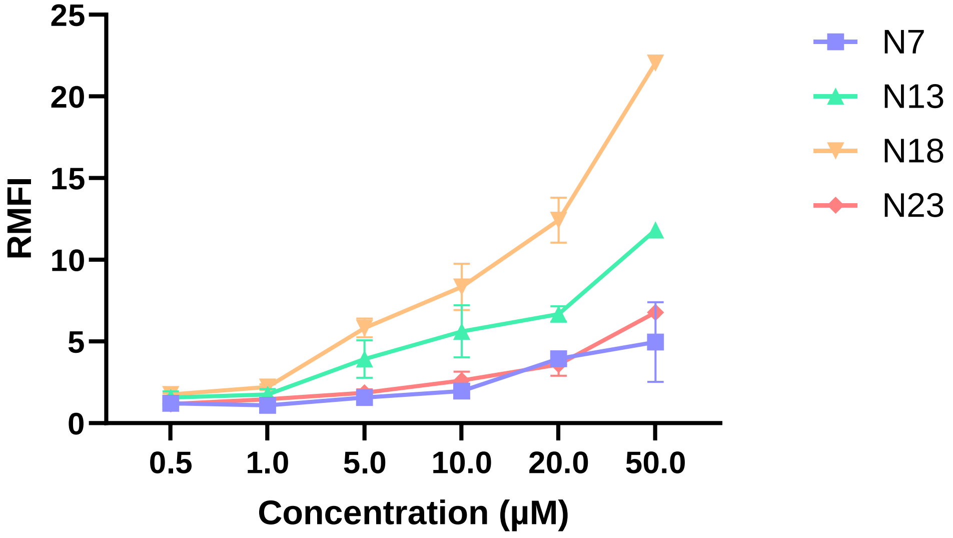

Figure S3: Binding of Affitins on MSLN-expressing cells. Cells were stained with a concentration range of Affitins, N1, N7, N13, N18 and N23 to validate their capacity to bind to MSLN-expressing cells. The binding was quantified by flow cytometry. Results are expressed as the mean of ratios of median intensities of fluorescence (RMFI) +/- SEM of three independent experiments.

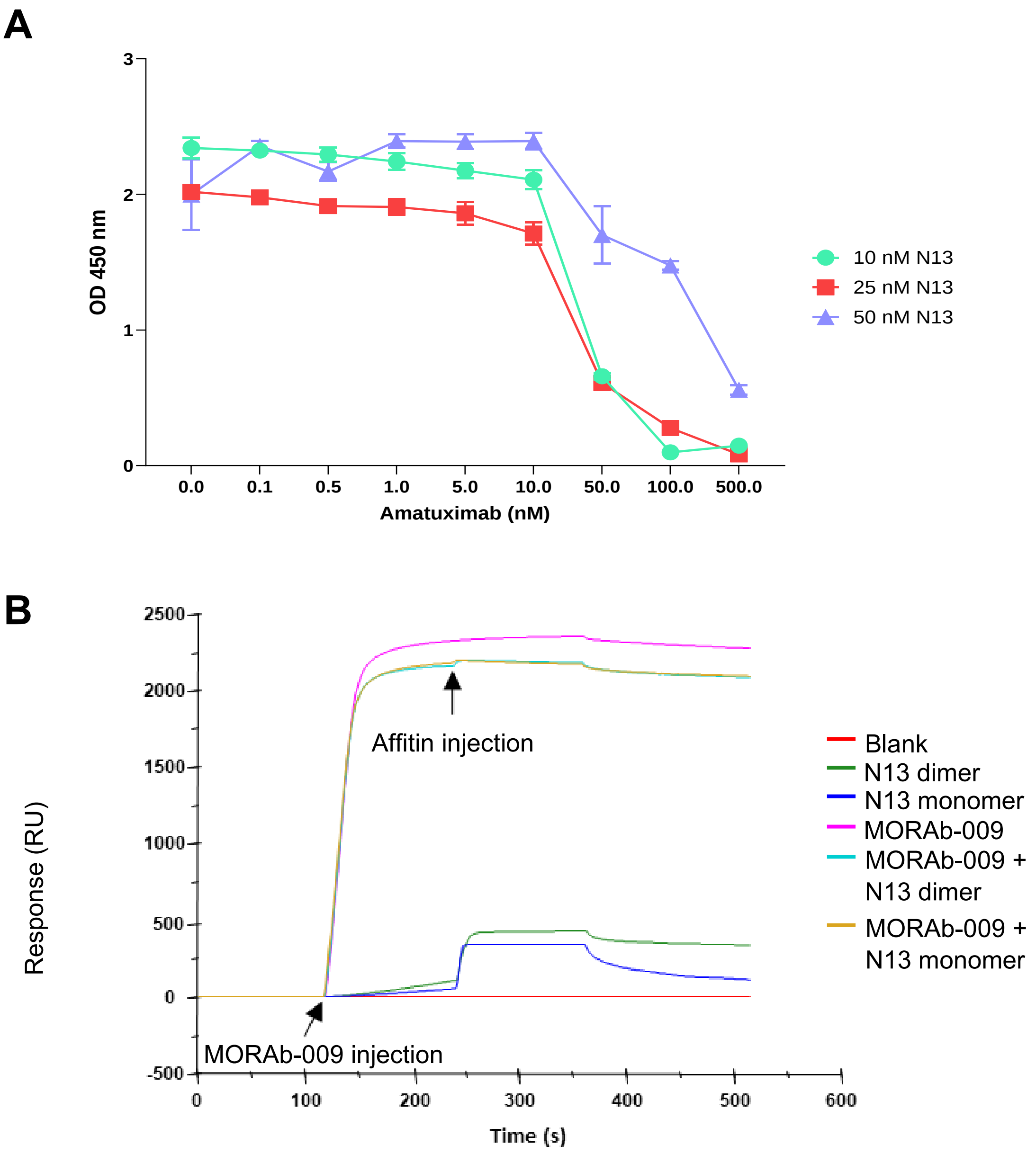

Figure S4: N13 and N13 dimer appear specific for the same mesothelin fragment as the MORAb-009 therapeutic antibody. The capacity of the N13 Affitin (A-B) and its homodimer (B) to compete with the MORAb-009 therapeutic antibody for their binding to human recombinant mesothelin fragment 302-359 was assessed by competitive ELISA (A) and surface plasmon resonance (B). A) biotinylated Mesothelin fragment was coated on wells of a 96-well maxisorp plate previously treated with neutravidin. Histidine-tagged N13 Affitin was added at different concentrations (10, 25 or 50 nM). Then, for each N13 Affitin conditions, increasing concentrations of MORAb-009 was added. N13 Affitin bound to MSLN fragment was revealed using anti-RGS-His6-HRP antibody. Results are means +/- SEM, n=3. OD, optical density. B) A first injection of a MORAb-009 solution was followed by a second injection of a mixed solution containing MORAb-009 and either the N13 monomer or the N13 dimer.

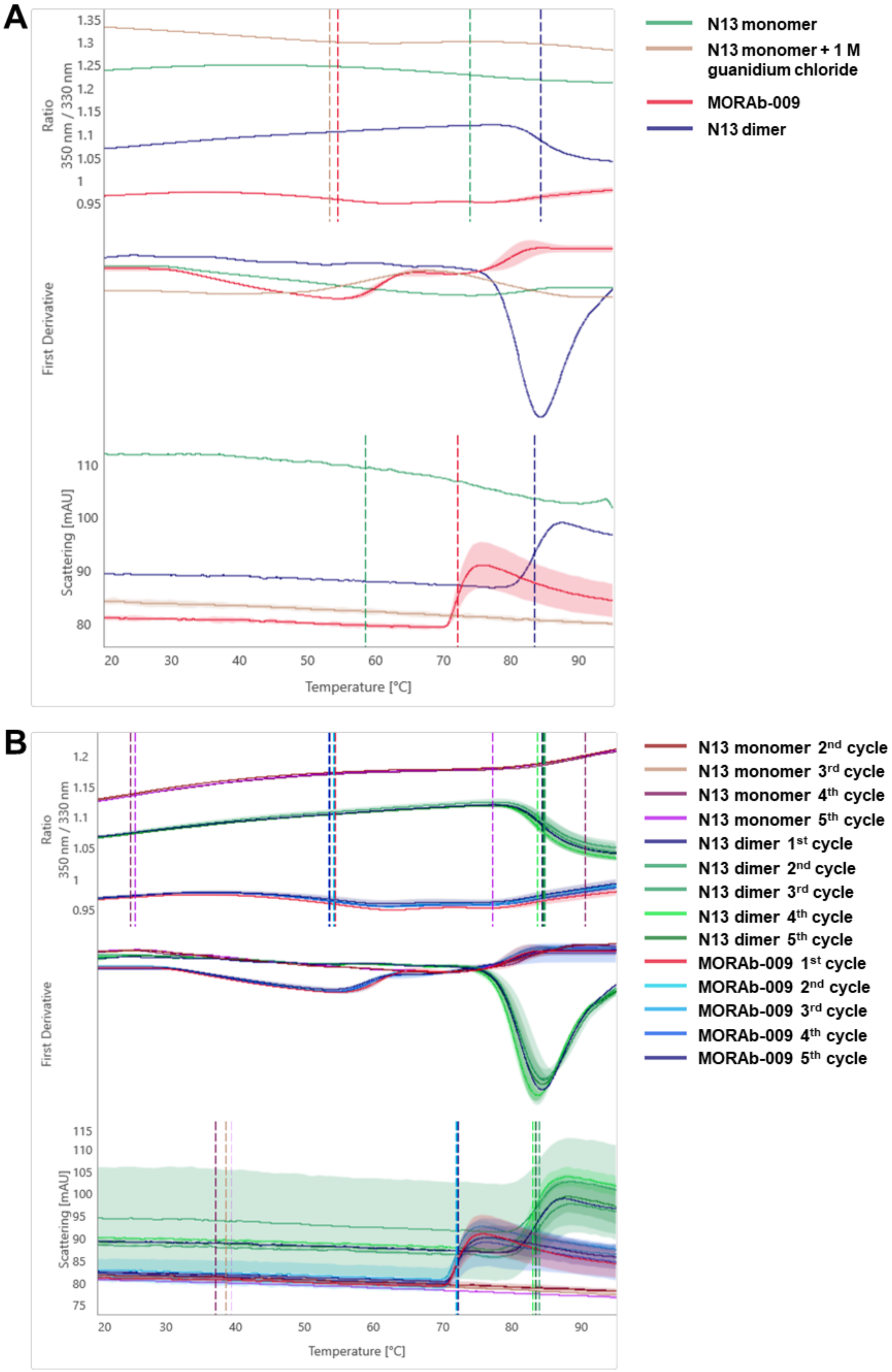
A
N13 monomer
N13 monomer + 1 M guanidium chloride
MORAb-009
N13 dimer
Ratio 350 nm / 330 nm
First Derivative
Scattering [mAU]
Temperature [°C]
B
N13 monomer 2nd cycle
N13 monomer 3rd cycle
N13 monomer 4th cycle
N13 monomer 5th cycle
N13 dimer 1st cycle
N13 dimer 2nd cycle
N13 dimer 3rd cycle
N13 dimer 4th cycle
N13 dimer 5th cycle
MORAb-009 1st cycle
MORAb-009 2nd cycle
MORAb-009 3rd cycle
MORAb-009 4th cycle
MORAb-009 5th cycle

Figure S5: N13 monomer and dimer are highly stable upon temperature variations and repeated freeze/thaw cycles. A) The thermal stability of the N13 monomer or dimer and of the MORAb-009 therapeutic antibody was assessed with the nanoDSF technology and are presented as NanoDSF, derivatives and scattering curves. B) The thermal stability of the N13 monomer or dimer and of the MORAb-009 therapeutic antibody was assessed with the nanoDSF technology after several freeze/thaw cycles. NanoDSF, derivatives and scattering curves are presented in (a).

A

	MORAb-009
ka1 (1/Ms)	1.27E+06
kd1 (1/s)	7.25E-04
ka2 (1/s)	37.46
kd2 (1/s)	20.48
Rmax (RU)	4324
K_D (M)	5.69E-10

B

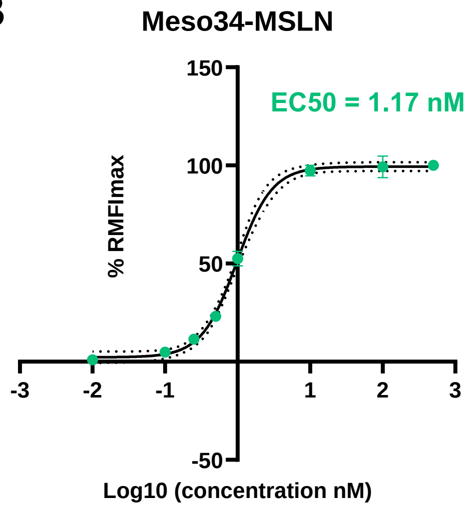

Figure S6: Characterization of the affinity of the MORAb-009 therapeutic antibody. A) Affinity constants of the MORAb-009 antibody obtained by surface plasmon resonance and calculated with the bivalent model. B) Meso34-MSLN (MSLN+) were stained with a concentration range of the MORAb-009 antibody and its binding was quantified by flow cytometry (n=3 independent experiments). Results are displayed as the means of ratios of median intensities of fluorescence (RMFI) +/- SEM of the samples normalized on median intensity of fluorescence of the secondary antibody alone.

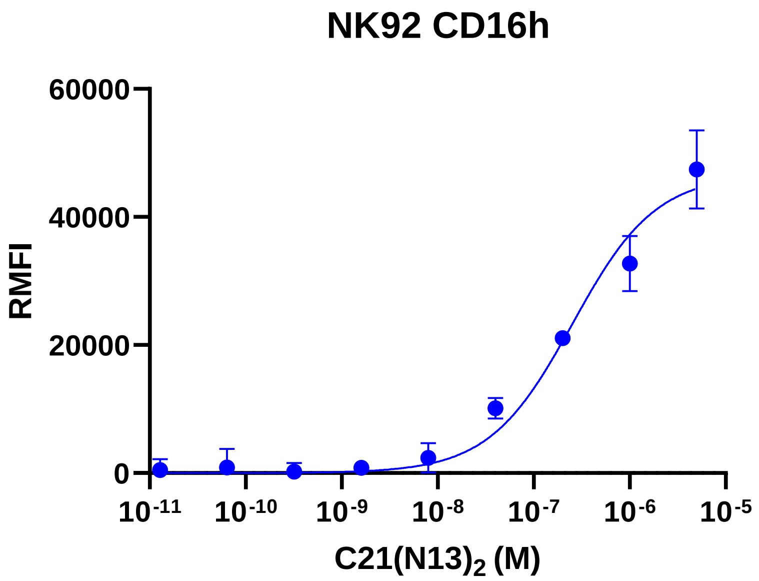

Figure S7: C21(N13)$_2$ NK cell engagers binding on NK-92CD16h cells. Bispecific NK engagers (BiKEs) composed of a dimer (C21(N13)$_2$) of the N13 Affitin fused to the C21 anti-CD16 nanobody were generated. NK92CD16h cells were stained with a concentration range of C21(N13)$_2$ BiKEs to validate the capacity of the constructions to bind to CD16 respectively. The binding was quantified by flow cytometry. Results are expressed as the mean of ratios of median intensities of fluorescence (RMFI) +/- SEM of two independent experiments.

A

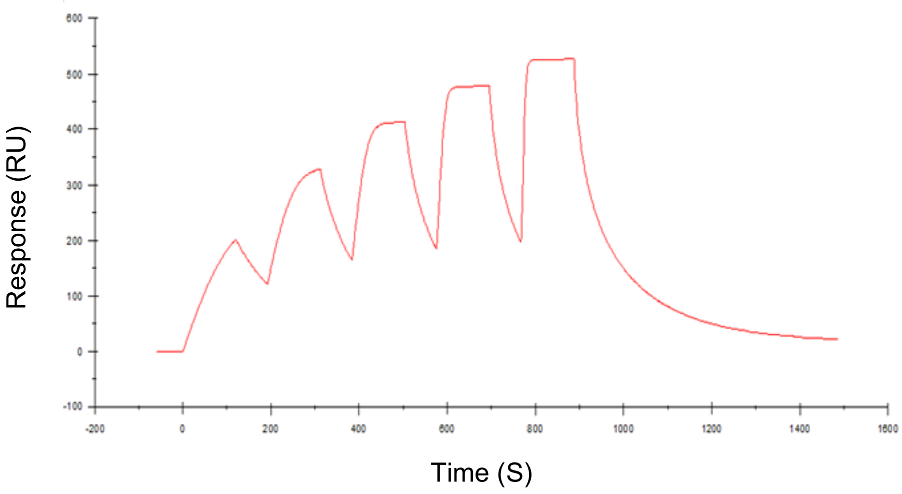

B

**ka (1/Ms)**	5.17E+07
**kd (1/s)**	8.04E-01
**Rmax (RU)**	556.7
**K_D (M)**	1.56E-08

Figure S8: Evaluation of VHH A1 affinity for recombinant hMSLN302-359 fragment. A) The capacity of VHH A1 to bind to human recombinant mesothelin fragment 302-359 was assessed by surface plasmon resonance. The affinity constants obtained, calculated with the bivalent model, are displayed in the table in (B).

Table S1: Sequences of proteins used in the study.

Protein	Sequence	e_m ($M^{-1}cm^{-1}$)*
hMSLN 302-359	MGSCPSGKKAREIDESLIFYKKWELEACVDAALLATQMDR VNAIPFTYEQLDVLKHKLDELKLGSGLNDIFEAQKIEWHEG HHHHHH	14180
Biotinylated $(N13)_1$	MRGSHHHHHHGSATKVKFKVFGEEKEVDISKIKHVDRMN WRGLLYIRFSYDDNGKLGHGWVSEKDAPKELLEKLKLGS GLNDIFEAQKIEWHE	19630
Biotinylated $(N13)_2$	MRGSHHHHHHGSATKVKFKVFGEEKEVDISKIKHVDRMN WRGLLYIRFSYDDNGKLGHGWVSEKDAPKELLEKLKPSGQ AGAAASESLFVSNHAYGSGATKVKFKVFGEEKEVDISKIKH VDRMNWRGLLYIRFSYDDNGKLGHGWVSEKDAPKELLEK LKLGSGLNDIFEAQKIEWHE	34850
N7	MRGSHHHHHHGSATKVKFKYMGEEKEVDISKIKVVSRSV GPLGSFILFQYDDNGKMGRGFVSEKDAPKELLEKLKLN	8250
N13	MRGSHHHHHHGSATKVKFKVFGEEKEVDISKIKHVDRMN WRGLLYIRFSYDDNGKLGHGWVSEKDAPKELLEKLKLN	13940
N18	MRGSHHHHHHGSATKVKFKLFGEEKEVDISKIKKVERRTF HMDKTIVFMYDDNGKSGYGWVSEKDAPKELLEKLKLN	8250
N23	MRGSHHHHHHGSATKVKFKLFGEEKEVDISKIKRVERRAI QAASYIAFAYDDNGKIGHGWVSEKDAPKELLEKLKLN	13940
Biotinylated-N13	MRGSHHHHHHGSATKVKFKVFGEEKEVDISKIKHVDRMN WRGLLYIRFSYDDNGKLGHGWVSEKDAPKELLEKLKLGS GLNDIFEAQKIEWHE	19630
Biotinylated-N18	MRGSHHHHHHGSATKVKFKLFGEEKEVDISKIKKVERRTF HMDKTIVFMYDDNGKSGYGWVSEKDAPKELLEKLKLGSG LNDIFEAQKIEWHE	13940
Biotinylated BiKE-$(N13)_1$	MEVQLVESGGELVQAGGSLRLSCAASGLTFSSYNMGWFR RAPGKEREFVASITWSGRDTFYADSVKGRFTISRDNAKNTV YLQMSSLKPEDTAVYYCAANPWPVAAPRSGTYWGQGTQV TVSSPSGQAGAAASESLFVSNHAYGSATKVKFKVFGEEKE VDISKIKHVDRMNWRGLLYIRFSYDDNGKLGHGWVSEKD APKELLEKLKKLNRGSHHHHHHGSGLNDIFEAQKIEWHE	51590
Biotinylated BiKE-$(N13)_2$	MEVQLVESGGELVQAGGSLRLSCAASGLTFSSYNMGWFR RAPGKEREFVASITWSGRDTFYADSVKGRFTISRDNAKNTV YLQMSSLKPEDTAVYYCAANPWPVAAPRSGTYWGQGTQV TVSSPSGQAGAAASESLFVSNHAYGSATKVKFKVFGEEKE VDISKIKHVDRMNWRGLLYIRFSYDDNGKLGHGWVSEKD APKELLEKLKPSGQAGAAASESLFVSNHAYGSGATKVKFK VFGEEKEVDISKIKHVDRMNWRGLLYIRFSYDDNGKLGHG WVSEKDAPKELLEKLKLNRGSHHHHHHGSGLNDIFEAQKI EWHE	66810
VHH A1	GSDVQLQESGGGLVHPGGSLRLSCAASGIDLSLYRMRWYR QAPGKERDLVALITDDGTSYYEDSVKGRFTITRDNPSNKVF LQMNSLKPEDTAVYYCNAETPLSPVNYWGQGTQVTVSSK LNRGSHHHHHH	20580

* Values were obtained using the VectorNTI Advance software (ThermoFisher)

Table S2: Thermal stability values upon repeated freeze/thaw cycles for N13 monomer, N13 dimer and MORAb-009 antibody.

Sample ID	Concentration	Initial value		Tm On		Tm 1		Tm2		Tm3		Agg On		IP Agg	
		Value	S.D.	Value	S.D.	Value	S.D.	Value	S.D.	Value	S.D.	Value	S.D.	Value	S.D.
**N13 monomer 1st cycle**	160 µg/mL	1.1462	0.0007	X	X	82.84	2.17	X	X	X	X	X	X	X	X
**N13 monomer 2nd cycle**	320 µg/mL	1.1456	0.0012	X	X	84.24	X	X	X	X	X	X	X	X	X
**N13 monomer 3rd cycle**	320 µg/mL	1.1429	0.0001	X	X	90.57	6.5	X	X	X	X	X	X	X	X
**N13 monomer 4th cycle**	320 µg/mL	1.1407	X	X	X	90.60	X	X	X	X	X	X	X	X	X
**N13 monomer 5th cycle**	320 µg/mL	1.1428	X	X	X	90.2	X	X	X	X	X	X	X	X	X
**N13 dimer 1st cycle**	1 mg/mL	1.0828	X	79.9	X	84.4	X	X	X	X	X	79.45	X	83.41	X
**N13 dimer 2nd cycle**	1 mg/mL	1.0812	0.0027	79.99	0.33	84.52	0.27	X	X	X	X	83.58	0.21	79.38	0.46
**N13 dimer 3rd cycle**	1 mg/mL	1.0805	0.0014	80.18	0.88	84.83	1.43	X	X	X	X	79.79	1.09	80.18	0.88
**N13 dimer 4th cycle**	1 mg/mL	1.079	0.0001	79.37	0.41	83.77	0.43	X	X	X	X	78.35	0.21	83.05	0.42
**N13 dimer 5th cycle**	1 mg/mL	1.0784	0.0006	79.84	0.66	84.48	1.00	X	X	X	X	79.56	0.83	83.49	0.67
**MORAb-009 1st cycle**	177 µg/mL	0.9763	0.0013	X	X	54.51	0.64	65.26	0.08	74.83	0.7	70.23	0.47	72.16	0.22
**MORAb-009 2nd cycle**	177 µg/mL	0.9795	0.0006	X	X	54.17	0.22	64.83	0.39	75.33	0.31	70.16	0.04	72.04	0.00
**MORAb-009 3rd cycle**	177 µg/mL	0.9789	0.0002	X	X	54.32	0.96	64.92	0.93	74.23	0.31	69.98	0.37	71.95	0.19
**MORAb-009 4th cycle**	177 µg/mL	0.9792	0.0004	X	X	53.6	0.96	64.55	1.09	75.22	0.47	70.32	0.32	72.17	0.26
**MORAb-009 5th cycle**	177 µg/mL	0.9800	0.0022	X	X	53.7	0.83	64.28	0.39	74.62	1.01	70.49	0.17	72.23	0.17

Selection of hMSLN-specific binders by ribosome display

Selections were carried-out at 4°C using the *in vitro* translated libraries of Affitins L5 and L6 as described in[10,56] with a few modifications regarding target presentation and the way the selected mRNAs were eluted. Briefly, 140 µL of translation mix[57] were incubated for 1 h at 4°C with shaking to allow binding of the Affitins-ribosome-mRNA ternary complexes to the target presented as follows. The first and third rounds of selection were performed using Dynabeads M270-Epoxy beads (25 µL) (Invitrogen) on which hMSLN was immobilized according to instructions provided by the manufacturer. For the second and fourth rounds of selection, biotinylated hMSLN 302-359 was used in solution at a concentration of 200 nM and 10 nM, respectively, and Affitins-ribosome-mRNA ternary complexes were captured using 50 µL of StreptaDivin beads (Ademtech) for 15 min with shaking. Prior the selection, the translation mix was pre-incubated with the corresponding beads in the absence of the target for 1 h and transferred to a new tube to get rid of bead-specific binders. Six washes for rounds 1 & 2, and eight washes for rounds 3 and 4, were performed using washing buffer (WBT, 50 mM Tris-acetate pH 7.4, 150 mM NaCl, 50 mM Magnesium acetate, 0.1% (v/v) Tween-20 (all reagents from Euromedex) to remove unbound complexes. For the first two rounds of selection, selected mRNAs were eluted using 200 µL of elution buffer (EB, 50 mM Tris-acetate pH 7.4, 150 mM NaCl, 20 mM ethylenediaminetetraacetic acid (EDTA) (all reagents from Euromedex)) to dissociate ribosomes for 10 min with shaking. For the third and fourth rounds, selected mRNA were eluted using either the EB buffer, or the VHH A1 at 10 µM (16 h with shaking) to specifically recover Affitins bound to the fragment recognized by A1. In order to obtain DNA from eluted mRNAs, a RT-PCR was performed at the end of the first round as follows: an initial denaturation step at 98°C for 30 s, followed by 35 cycles of 10 s at 98°C, 30 s at 61°C, and 10 s at 72°C with a final elongation step of 5 min at 72°C. For the following rounds, the number

of cycles were as follows: 30 cycles for round 2, for round 3 when elution was performed with EB and for round 4 when elution was performed with A1 or 35 cycles for round 3 when elution was performed with A1, and 25 cycles for round 4 when elution was performed with EB. The primers used for the RT-PCR (Eurofins) were RDV2.1-F3 (5'-GATGACGATGACAAAGGATCC-3') and AG1-link-R (5'-GAATTCGGCCCCCGAGGCCATATAAAGC-3'). The general strategy used for Affitins selection is summarized in fig. S1.

Clusterization of NGS data

DNA sequences obtained from the different selection rounds were first pooled and translated into proteins. Sequences containing an internal stop codon were considered as contaminant and removed from the analysis. This step removed between 20 and 30 percent of DNA sequences depending on the selection round. Redundant sequences were also removed. Then, a consensus sequence was calculated as the sequence with the lower distance to the whole dataset according to the Grantham matrix[58]. The Grantham matrix was chosen because of its ease of use in quantifying the difference between two amino acids according to their composition, polarity, and molecular volume. Each amino acid for each unique sequence was translated into a number corresponding to their distance to the consensus sequence according to the Grantham matrix. This allows to consider every amino-acid position as a quantitative parameter. Dimensionality reduction was then performed by principal component analysis (PCA) to speed up further clustering (80% of variance explained). Clustering was performed using density-based spatial clustering of applications with noise (DBSCAN) algorithm with the ε value set as 10 and the minimum sample set as 250. We obtained 82 clusters of sequences, for which a consensus sequence was then calculated.

Production of the VHH A1

The nucleotide sequence of the VHH A1[22] was synthetized and inserted via BamHI and HindIII restriction sites in pFP4101 plasmid, a derivate of the pQe30 vector (Quiagen) enabling the expression of proteins in the periplasm of *E. coli* thanks to a dsbA leader sequence and inserting an hexa histidine tag at their N-terminal. This plasmid was used to transform the *E. coli* DH5α I^q strain (Thermo Fisher Scientific) and the expression of the VHH A1 was induced with 0.5 mM IPTG (Euromedex) at 25°C for 64 h. Bacteria were harvested by centrifugation and the pellet was resuspended in ice-cold buffer (TES, 0.2 M Tris pH 8, 0.5 mM EDTA, 0.5 M sucrose (all reagents from Euromedex)). After a four folds dilution with ice-cold water to induce an osmotic shock, the bacterial suspension was incubated for 30 min on ice with gentle agitation. The concentrations of NaCl and Imidazole (Euromedex) were adjusted to 500 mM and 20 mM, respectively. After centrifugation at 8 500 g for 40 min at 4°C, the VHH A1 contained in the periplasmic extract was purified using a Ni-NTA resin (Qiagen) and size-exclusion chromatography on a Superdex 75 gel filtration column (Cytiva) equilibrated with PBS 7.4.

Nano differential scanning fluorimetry (NanoDSF)

a) Thermal stability

Ten microliters of samples were loaded into nanoDSF grade standard or high sensitivity capillaries (NanoTemper Technologies, Munich, Germany), for samples with a concentration higher than 1 mg/mL or lower than 500 µg/mL respectively, and installed on capillary array on the Prometheus NT.48 nanoDSF instrument (NanoTemper Technologies, Munich, Germany). For the guanidium chloride conditions, the N13 Affitin was incubated for 2 h at room temperature with 1 M guanidium chloride prior to the injection in the capillaries. The temperature was increased from 20 °C to 95

°C with a linear thermal ramp (at a 1 °C/min rate). Changes in fluorescence of tryptophan at 330 and 350 nm due to denaturation of the proteins were recorded at 10 datapoints per minute rate. The 350/330 nm ratio of the two wavelengths were plotted against temperature and the first derivative analysis allowed the determination of Tm using the PR. ThermControl Software (NanoTemper Technologies, Munich, Germany). The Prometheus NT.48 also monitors the aggregation of the samples during heating.

b) Stability to repeated freeze/thaw cycles

Samples were subjected to several freeze-thaw cycles of 1 hour at -80°C before they were analyzed on a Prometheus NT.48 nanoDSF instrument (NanoTemper Technologies, Munich, Germany) as described above.

Surface Plasmon Resonance (SPR): Competition experiments

Pair-wise fragment mapping was performed on a CM5 chip functionalized with recombinant hMSLN 302-359 as described above (level of immobilization: 700 RU). The measurements were carried out at 25°C with a 30 μL/min flow rate using the Dual Inject method. Briefly, a first injection of a 100 nM solution of MORAb-009 antibody (Proteogenix PX-TA1049) for 2 min was followed by a 2 min second injection of a mixed solution containing 100 nM of MORAb-009 antibody and either 500 nM of N13 monomer or 500 nM of N13 dimer. Finally, after one min of dissociation of the complexes, a regeneration step was enabled by a 30 s pulse injection of regeneration solution (10 mM NaOH) to remove the captured proteins.
